# Pesticide Residues in Fresh Vegetables in Türkiye: Regulatory Compliance, Risk Ranking, and Deterministic and Probabilistic Dietary Risk Assessment

**DOI:** 10.3390/foods15213898

**Published:** 2026-10-30

**Authors:** Sebahat Oztekin, Eylem Odabas, Ozgur Golge, Miguel Ángel González-Curbelo, Bulent Kabak

**Affiliations:** 1 Department of Food Engineering, Faculty of Agriculture, Ataturk University, Erzurum 25240, Türkiye; sebahat.oztekin@atauni.edu.tr; 2 Department of Food Engineering, Faculty of Engineering and Natural Sciences, Hitit University, Corum 19030, Türkiye; eylem.odabas@ogrenci.hitit.edu.tr; 3 Department of Gastronomy and Culinary Arts, Faculty of Tourism, Alanya Alaaddin Keykubat University, Alanya 07425, Türkiye; ozgur.golge@alanya.edu.tr; 4 Departamento de Ciencias Básicas, Facultad de Ingeniería, Universidad EAN, Calle 79 n11-45, Bogotá 110221, DC, Colombia; magonzalez@universidadean.edu.co

**Keywords:** pesticide residues, pepper, cucumber, tomatoes, courgette, dietary exposure, Monte Carlo simulation, Index of Quality for Residues (IqR)

## Abstract

Pesticide residues in fresh vegetables require comprehensive evaluation to determine their regulatory compliance, relative priority, and potential dietary health implications. In this study, 300 sweet pepper, cucumber, tomato, and courgette samples (*n* = 75 each) collected in Türkiye were analysed for 218 pesticides using the quick, easy, cheap, effective, rugged, and safe (QuEChERS) extraction method coupled with liquid chromatography-high-resolution-tandem mass spectrometry (LC-HRMS/MS). Quantifiable residues were found in 31% of samples, with detection frequencies of 49.3% in pepper, 34.7% in cucumber, 32% in tomato, and 8% in courgette. Nineteen samples (6.3%) contained residues exceeding European Union (EU) maximum residue levels, with cucumber showing the highest exceedance rate (17.3%). Residue quality was further characterised using the Index of Quality for Residues (*IqR*), while a risk-ranking approach identified acetamiprid as the highest-priority pesticide, particularly in cucumber and pepper. Deterministic assessment indicated low chronic dietary concern for both adults and children. Potential acute concern was identified for acetamiprid in three cucumber samples for adults and 11 for children, and in two tomato samples for children. Probabilistic assessment using Monte Carlo simulation supported the chronic findings, with all 95th percentile hazard index values remaining below 1, including under the combined-consumption scenario. These findings demonstrate the value of integrating residue occurrence, regulatory compliance, residue quality, risk prioritisation, and dietary exposure assessment for pesticide residue surveillance.

## 1. Introduction

Pesticides are widely used in agricultural production to control pests, diseases, and weeds, thereby reducing crop losses and helping to maintain the quality and marketability of agricultural commodities. Their use, however, may result in the presence of residues in harvested products, particularly when application rates, treatment frequencies, or pre-harvest intervals are not adequately observed [1]. Although pesticide residues do not necessarily indicate a health risk, their occurrence in food requires careful evaluation because the potential for adverse effects depends on the toxicological properties of the active substance and the magnitude and duration of exposure [2]. Long-term exposure to certain pesticides has been associated with neurotoxic, endocrine, reproductive, genotoxic, and carcinogenic effects, although these outcomes are substance- and exposure-dependent [3]. A comprehensive evaluation of pesticide residues therefore requires consideration of both regulatory compliance and the potential implications of dietary exposure.

Maximum residue levels (MRLs) established by the European Union (EU) provide a regulatory basis for assessing pesticide residues in food commodities. Compliance with these limits is an important component of food control, but MRLs should not be interpreted as direct thresholds of toxicological safety. Dietary risk assessment provides a complementary perspective by relating estimated exposure to toxicological reference values, thereby allowing the potential health relevance of detected residues to be evaluated. Alongside regulatory controls, sustainable agricultural practices and Integrated Pest Management (IPM) have been promoted to reduce reliance on chemical pesticides [4]. Pesticide residues nevertheless remain a prominent concern in food monitoring, as reflected in the 6239 notifications recorded in the Rapid Alert System for Food and Feed (RASFF) between January 2020 and August 2026, of which 3605 were associated with fruits and vegetables [5].

Vegetables are an essential component of a balanced diet, providing dietary fibre, vitamins, minerals, and a wide range of bioactive compounds. Regular vegetable consumption has been associated with a lower incidence of several non-communicable diseases, including cardiovascular diseases and certain cancers [6]. Among commonly consumed vegetables, sweet pepper (*Capsicum annuum* L.), cucumber (*Cucumis sativus* L.), tomato (*Solanum lycopersicum* L.), and courgette (also known as zucchini, *Cucurbita pepo* L.) are important both as fresh foods and as ingredients in a wide range of domestic and industrial food preparations. Their production is subject to pressure from fungal, oomycete, bacterial, and viral diseases, as well as insect and mite pests, which may necessitate the application of different pesticide classes. Among the diseases affecting these crops, *Phytophthora capsici* in pepper, early and late blight in tomato, and downy and powdery mildew in cucurbits are of particular importance. Aphids, thrips, whiteflies, and mites also affect these crops, while the tomato leafminer (*Tuta absoluta*) represents a major pest in tomato production [7,8,9]. These production challenges, together with differences in crop protection practices, may contribute to variation in pesticide residue profiles among vegetable commodities.

Pesticide residues in pepper, cucumber, tomato, and courgette have been investigated in different geographical regions, with studies reporting variation in residue occurrence, pesticide diversity, and regulatory compliance [10,11,12,13,14,15]. These variations may reflect differences in agricultural practices, production systems, pesticide authorisations, and sampling periods. However, residue occurrence and MRL compliance alone do not fully characterise the potential significance of pesticide contamination. A more comprehensive evaluation requires consideration of multiple-residue occurrence, the relative priority of individual pesticides, and dietary exposure across different population groups. The index of quality for residues (*IqR*) provides an additional indicator for assessing pesticide residue levels in relation to established MRLs, while risk-ranking approaches can assist in identifying pesticides that warrant greater attention [15]. Deterministic and probabilistic exposure assessments provide complementary estimates of potential health risks, with probabilistic modelling accounting for variability in residue concentrations and food consumption. However, the combined application of these approaches across multiple vegetable commodities remains insufficiently explored, particularly in Türkiye.

The present study therefore aimed to provide an integrated evaluation of pesticide residues in four commonly consumed vegetables marketed in Türkiye. Pesticide residues in pepper, cucumber, tomato, and courgette samples were determined, and the detected concentrations were assessed against the relevant EU MRLs. Sample quality was evaluated using the *IqR*, and the detected pesticides were prioritised using a risk-ranking approach. Chronic and acute dietary exposure and associated health risks were evaluated for adults and children at individual and cumulative levels using deterministic approaches. In addition, a probabilistic assessment based on Monte Carlo simulation (MCS) was performed to incorporate variability in residue concentrations and consumption amounts into exposure estimates. This comprehensive approach aimed to determine whether differences in residue occurrence and regulatory compliance were reflected in dietary risk estimates and to identify the pesticides and commodities warranting greater attention in risk-based surveillance.

## 2. Materials and Methods

### 2.1. Chemicals and Reagents

Analytical reference standards for the 218 target pesticides, with purities exceeding 95%, were obtained from CPAchem (Sofia, Bulgaria), Dr. Ehrenstorfer GmbH (Augsburg, Germany), and Sigma-Aldrich (Steinheim, Germany). Individual stock solutions of each pesticide were prepared at 1000 mg L^−1^ using water, methanol, acetonitrile, or acetone, as appropriate according to the physicochemical properties and solubility of each analyte. The stock solutions were transferred to amber glass vials and stored at −18 °C until use. A mixed intermediate standard solution containing all target pesticides at 10 µg mL^−1^ was prepared in acetonitrile and used to prepare matrix-matched calibration standards and fortification solutions for method validation.

LC-MS-grade methanol and acetonitrile were obtained from Sigma-Aldrich (Steinheim, Germany). Ammonium formate, formic acid, and analytical-grade glacial acetic acid (CH_3_COOH, 100%) were purchased from Merck KGaA (Darmstadt, Germany), and ultrapure water was produced using a Milli-Q purification system (Millipore, Molsheim, France). Anhydrous magnesium sulphate (MgSO_4_) and sodium acetate (CH_3_COONa) were obtained from Carlo Erba Reagents (Val-de-Reuil, France), while primary-secondary amine (PSA) sorbent was supplied by Supelco (Bellefonte, PA, USA). Polytetrafluoroethylene (PTFE) membrane filters (0.22 µm) were obtained from Whatman (Clifton, NJ, USA).

### 2.2. Sample Collection and Sample Preparation

A total of 300 fresh vegetable samples, comprising sweet pepper (*n* = 75), cucumber (*n* = 75), tomato (*n* = 75), and courgette (*n* = 75), were collected from 18 supermarkets in Alanya, Antalya, and Çorum, Türkiye, between May and August 2026. Samples were randomly selected from the vegetables at the time of purchase, with approximately 2 kg obtained per sample. Following transportation to the laboratory, stems were removed and the whole commodities were homogenised without washing or peeling using a laboratory homogeniser (Retsch GM 300, Haan, Germany) prior to pesticide residue extraction.

**Figure 1:**
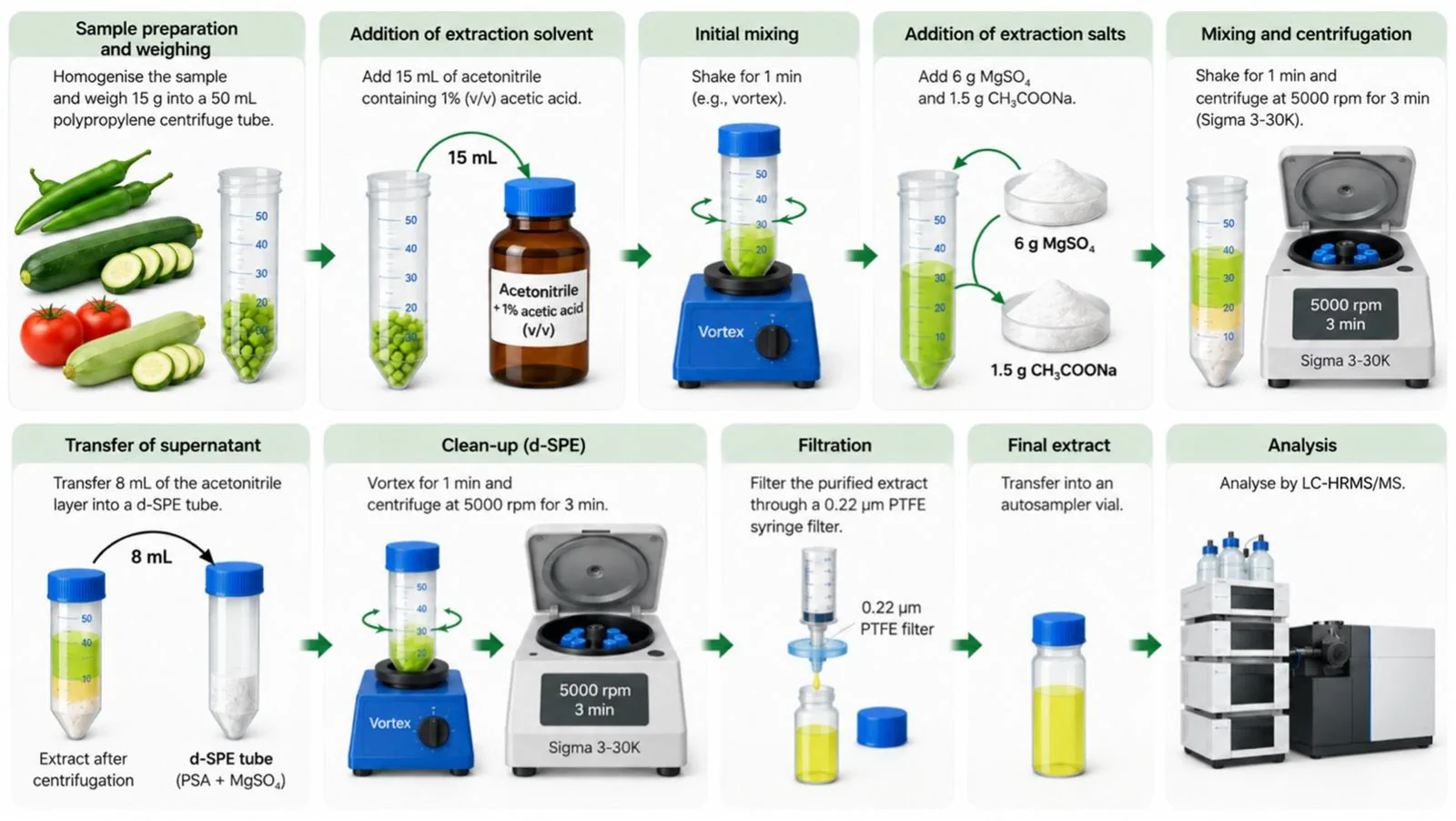
Schematic representation of the QuEChERS-based workflow for sample preparation in pesticide residue analysis of vegetable samples.

Pesticide residues were extracted using the quick, easy, cheap, effective, rugged, and safe (QuEChERS) procedure in accordance with AOAC Official Method 2007.01 [16]. The procedure involved acetonitrile extraction followed by partitioning with anhydrous MgSO_4_ and CH_3_COONa and dispersive solid-phase extraction (d-SPE) clean-up using PSA and MgSO_4_. The sample preparation workflow is shown in Figure 1.

### 2.3. Instrumental Analysis

The 218 target pesticides were analysed using a Vanquish ultra-high-performance liquid chromatography (UHPLC) system coupled to a Q Exactive Focus Orbitrap high-resolution mass spectrometer (Thermo Fisher Scientific, Bremen, Germany). Chromatographic separation was achieved on an Accucore Vanquish C18 column (2.1 × 100 mm, 1.5 µm; Thermo Fisher Scientific) using gradient elution. Mobile phase A consisted of water–methanol (98:2, *v*/*v*) containing 0.1% formic acid and 5 mM ammonium formate, while mobile phase B consisted of methanol. The 13-min gradient programme was initiated at 80% A and 20% B. Mobile phase B was increased linearly to 70% within 1.5 min and maintained at this proportion until 10 min. The initial mobile phase composition was restored within 0.2 min, followed by column re-equilibration for the remainder of the run. The flow rate was 0.6 mL min^−1^, the column temperature was maintained at 40 °C, and the injection volume was 10 µL.

Mass spectrometric detection was performed using a heated electrospray ionisation (HESI) source. The spray voltage was set at 2.8 kV, with a source temperature of 320 °C and a vaporiser temperature of 295 °C. TraceFinder software (version 5.1; Thermo Fisher Scientific) was used for instrument control, data acquisition, and processing. Pesticides were identified and confirmed on the basis of chromatographic retention time, accurate mass, and at least two diagnostic product ions in accordance with SANTE/11312/2021 v2 [17]. The precursor and product ions used for identification and quantification of the target pesticides are provided in Table S1.

Method performance was evaluated in accordance with SANTE/11312/2021 v2 [17]. Pepper, cucumber, tomato, and courgette were classified as high-water-content commodities, with tomato selected as the representative matrix for method validation. Matrix-matched calibration standards were prepared by fortifying blank tomato extracts with the pesticide standards at five concentration levels ranging from 0.005 to 0.25 mg kg^−1^. Samples yielding analytical responses above the upper limit of the matrix-matched calibration range were diluted with blank matrix extracts and reanalysed to ensure quantification within the established calibration range. Matrix-matched calibration was used to minimize matrix-related effects during quantification. However, matrix effects were not independently evaluated for pepper, cucumber, and courgette, which may introduce additional uncertainty into pesticide quantification in these commodities. Linearity was assessed from the coefficients of determination (*R*^2^) of the matrix-matched calibration curves, and the limit of quantification (LOQ) was established as the lowest concentration meeting the applicable identification and quantitative performance criteria.

Trueness was assessed through recovery experiments at fortification levels of 0.010 and 0.050 mg kg^−1^, with five replicate analyses performed at each level (*n* = 5). Precision was evaluated in terms of repeatability (RSD_r_) and within-laboratory reproducibility (RSD_wR_). Repeatability was determined from the replicate analyses performed under the same analytical conditions, whereas within-laboratory reproducibility was evaluated using 10 replicate analyses (*n* = 10). Expanded measurement uncertainty was estimated from bias and within-laboratory reproducibility. Method performance was evaluated against the acceptance criteria specified in SANTE/11312/2021 v2, including recoveries of 70–120% and RSD_r_ and RSD_wR_ values ≤ 20%.

### 2.4. Regulatory Compliance and Residue Quality Assessment Using the IqR

Regulatory compliance was assessed by comparing quantified pesticide residues with the corresponding commodity-specific EU MRLs obtained from the EU Pesticides Database. The EU approval status of detected active substances was evaluated separately using the active substances section of the same database. Results below the validated LOQ were not classified as MRL exceedances.

Residue quality was assessed for each vegetable sample using the *IqR*, according to the approach described by Mac Loughlin et al. [18]. The *IqR* was calculated using Equation (1):

(1) $$\mathrm{IqR}\;=\sum_{i=0}^{n}\frac{\mathrm{PRCi}}{\mathrm{MRLi}}$$

where *PRC* represents the concentration of each pesticide residue detected in the sample, MRL is the corresponding maximum residue level, and *n* represents the number of pesticide residues detected in that sample. Based on the calculated *IqR* values, samples were classified into four residue-quality categories: excellent (*IqR* = 0), good (0 < *IqR* ≤ 0.6), adequate (0.6 < *IqR* ≤ 1.0), and inadequate (*IqR* > 1.0), according to the classification proposed by Mac Loughlin et al. [18].

### 2.5. Health Risk Assessment

#### 2.5.1. Risk Ranking

A matrix-based risk-ranking approach, adapted from the framework developed by the UK Veterinary Residues Committee [19], was used to establish the relative priority of the pesticide residues detected in the vegetable samples. Six indices reflecting toxicological and exposure-related factors were incorporated into the composite risk score (S): acute toxicity (A), toxicological potency (B), dietary contribution (C), pesticide application frequency (D), susceptibility of vulnerable population groups (E), and residue occurrence relative to the applicable maximum residue levels (F). The criteria used to assign scores to each index were based on the original ranking matrix and are presented in Table S2, while the scores assigned to indices A–F for the detected pesticides are provided in Table S3. The composite risk score was calculated using Equation (2):

*S* = (*A* + *B*) × (*C* + *D* + *E*) × *F* (2)

Index A was assigned on the basis of median lethal dose (LD_50_) values as an indicator of acute toxicity [20], whereas index B was derived from acceptable daily intake (ADI) values as an indicator of toxicological potency [21]. Dietary contribution (C) was determined using consumption data for pepper, cucumber, tomato, and courgette [22]. Index D reflected pesticide application frequency relative to the vegetable growth period. In the absence of sufficient evidence to identify population groups with particularly high exposure, the maximum score of 3 was assigned to index E, following the precautionary approach recommended by the UK Veterinary Residues Committee. Index F reflected the occurrence of pesticide residues relative to their corresponding MRLs.

Pesticide application frequency was characterised using the frequency of dosing (FOD), calculated as the number of pesticide applications (N) divided by the duration of the vegetable growth period (P, days), as shown in Equation (3):

(3) $$\mathrm{FOD}=\;\frac{N}{P}\;\times \;100$$

The residue occurrence index (F) was calculated from the distribution of analytical results among concentration categories defined relative to the applicable MRLs, using Equation (4):

(4) $$F=\frac{\left(F_{0}\;\times \;1)\;+\;{(F}_{1}\times \;2)\;+\;{(F}_{2}\;\times \;3)+\;{(F}_{3}\;\times \;4\right)}{n}$$

where F_0_ denotes the number of samples in which no residue of the pesticide was detected; F_1_, the number of samples with residue concentrations below the MRL; F_2_, the number of samples with concentrations ranging from one to ten times the MRL; and F_3_, the number of samples with concentrations at least ten times the MRL. The total number of samples included in the assessment is denoted by n.

#### 2.5.2. Deterministic Dietary Exposure and Risk Assessment

The national estimated daily intake (NEDI) and chronic hazard quotient (*HQc*) for each pesticide were calculated separately for adults and children aged 3–10 years using Equations (5) and (6), respectively:

(5) $$\mathrm{NEDI}=\frac{\mathrm{Residue}\;\mathrm{concentration}\;(\mathrm{mg}/\mathrm{kg})\;\times \;\mathrm{Consumption}\;\mathrm{rate}\;(\mathrm{kg})}{\mathrm{Body}\;\mathrm{weight}\;(\mathrm{kg})}$$

(6) $$\mathrm{HQc}=\frac{\mathrm{NEDI}}{\mathrm{ADI}}$$

Mean daily per capita consumption was 0.088 kg for pepper, 0.042 kg for cucumber, 0.341 kg for tomato, and 0.014 kg for courgette [22]. For children, daily consumption was assumed to correspond to 40% of the respective adult consumption amounts [23]. Body weights of 70 kg for adults and 23 kg for children were used [24]. To account for uncertainty associated with left-censored data, results below the LOQ were treated using the substitution approach recommended by EFSA [25]. Non-quantified results were assigned values of zero, LOQ/2, and LOQ under the lower-bound (LB), middle-bound (MB), and upper-bound (UB) scenarios, respectively.

Acute dietary exposure was estimated using the international estimated short-term intake (IESTI), and the associated acute hazard quotient (*HQa*) was subsequently calculated. IESTI estimates were derived using Cases 2a and 2b, as defined by WHO/FAO [26], according to Equations (7) and (8), while *HQa* was calculated using Equation (9):

Case 2a: (25 g ≤ *Ue* < *LP*)

(7) $$\mathrm{IESTI}=\frac{\mathrm{Ue}\times \mathrm{HR}\times v+\left(\mathrm{LP}-\mathrm{Ue}\right)\times \mathrm{HR}}{\mathrm{bw}}$$

Case 2b: (*LP < Ue*)

(8) IESTI=LP × HR × v bw

(9) $$\mathrm{HQa}=\frac{\mathrm{IESTI}}{\mathrm{ARfD}}$$

The unit weights of the edible portion (*Ue*) were 0.170 kg for pepper, 0.458 kg for cucumber, 0.175 kg for tomato, and 0.328 kg for courgette. For each pesticide–vegetable combination, the highest residue (HR) was defined as the maximum concentration measured among the 75 independently collected and homogenised samples of the respective vegetable, and a variability factor (*v*) of 3 was applied. Large portion (*LP*) consumption values for adults were 0.280 kg for pepper, 0.424 kg for cucumber, 0.377 kg for tomato, and 0.068 kg for courgette. For children, *LP* values were 0.169 kg for pepper, 0.212 kg for cucumber, 0.263 kg for tomato, and 0.021 kg for courgette [27]. ADI and acute reference dose (ARfD) values for each pesticide were retrieved from the EU Pesticides Database [21]. Where no ARfD had been established (ametoctradin, azoxystrobin, boscalid, chlorantraniliprole, clofentezine, cyantraniliprole, and metrafenone), the ADI was conservatively used as the reference value for acute exposure assessment, following the approach used in EFSA pesticide residue monitoring reports [28].

Cumulative chronic and acute risks were characterised using the chronic hazard index (*HIc*) and acute hazard index (*HIa*), respectively, calculated as the sums of the corresponding individual pesticide *HQs* according to Equation (10):

*HI* = *Σ HQ* (10)

The summed hazard indices were used as screening-level indicators of combined pesticide exposure, without assuming a common toxicological mode of action or dose additivity among the detected pesticides. *HQc*, *HQa*, *HIc*, and *HIa* values ≤ 1 were considered to indicate no appreciable health risk under the assessed exposure conditions, whereas values >1 indicated that the corresponding toxicological reference value was exceeded and that a potential health concern could not be excluded.

#### 2.5.3. Probabilistic Risk Assessment Using Monte Carlo Simulation

A probabilistic risk assessment was conducted using MCS to characterise variability in chronic dietary exposure and associated health risks. The simulation was performed in Microsoft Excel^®^ integrated with Oracle Crystal Ball^®^ software (version 11.1.34190, Oracle, Austin, TX, USA), with 10,000 iterations conducted for each exposure scenario. A log-normal distribution was assigned to residue concentrations to account for their right-skewed distribution, whereas body weight was modelled using a normal distribution. For the probabilistic assessment, non-detects and residue concentrations below the LOQ were assigned the corresponding LOQ value according to the UB approach. These inputs were propagated through the model to generate probabilistic distributions of the *HIc* for adults and children. The resulting distributions were characterised using the 5th, 50th, and 95th percentiles (P5, P50, and P95, respectively). Chronic risk was evaluated for each vegetable individually and for the combined consumption of all four vegetables. Sensitivity analysis (SA) was performed within the MCS framework to quantify the relative contributions of individual pesticides and vegetable commodities to the variance in the *HIc* for the combined consumption of all four vegetables.

## 3. Results and Discussion

### 3.1. Method Validation Results

**Figure 2:**
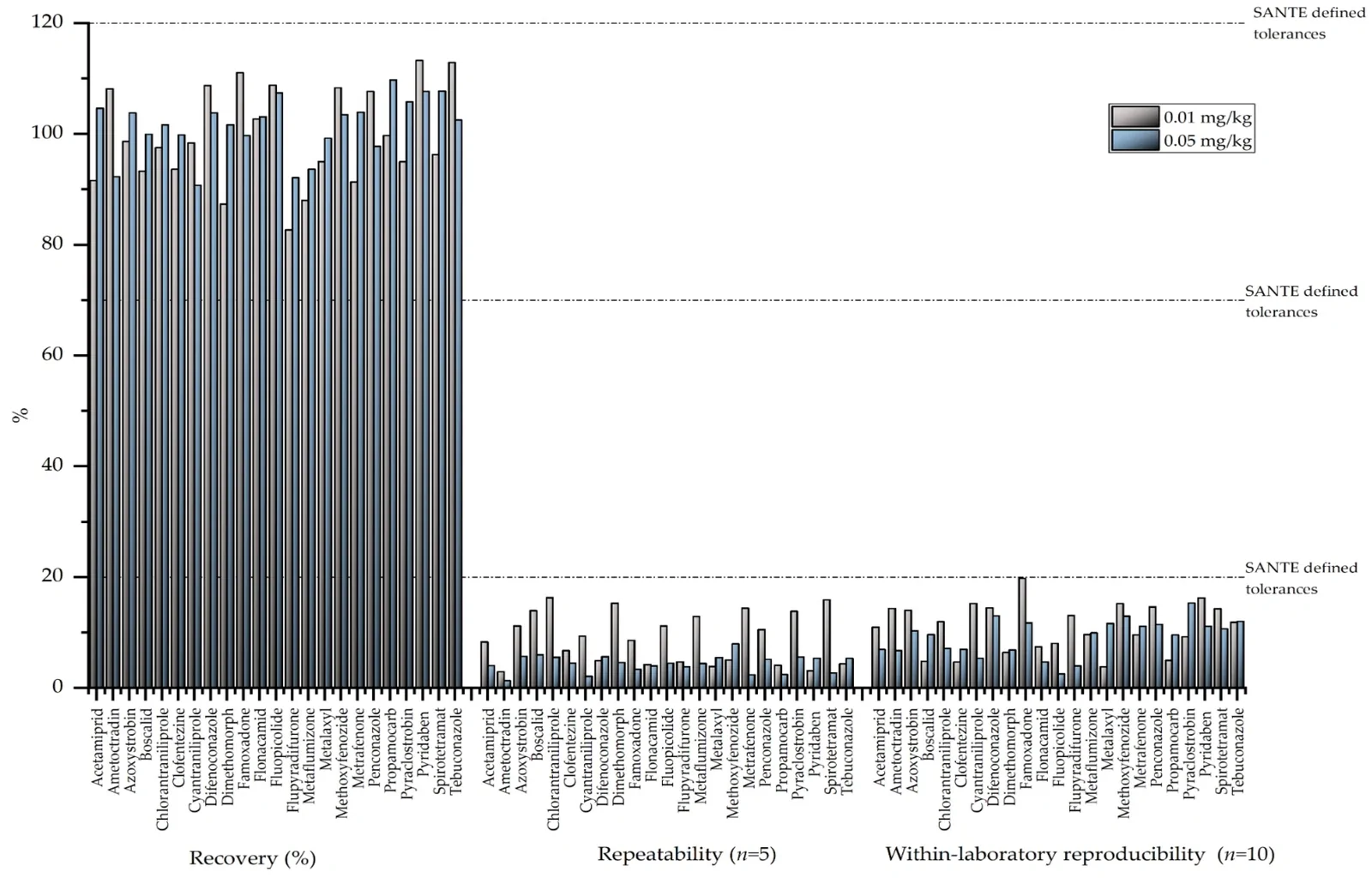
Recovery, repeatability, and within-laboratory reproducibility results for the detected pesticides during method validation. Dashed lines indicate the acceptance limits specified in SANTE/11312/2021 v2.

The analytical method showed satisfactory performance for the determination of pesticide residues in the vegetable matrices. *R*^2^ values exceeded 0.99 for all target analytes, and an LOQ of 0.01 mg kg^−1^ was achieved for each pesticide. For the pesticides detected in the analysed samples, mean recoveries at the two fortification levels ranged from 82.7 to 113.3%, with all values falling within the 70–120% acceptance range specified in SANTE/11312/2021 v2 (Figure 2). RSD_r_ and RSD_wR_ values ranged from 1.3 to 16.3% and from 2.5 to 19.7%, respectively, with all values remaining below the 20% acceptance limit. Expanded measurement uncertainty ranged from 13 to 30%. These results demonstrate that the method provided satisfactory trueness and precision for the determination of pesticide residues in the matrices evaluated.

### 3.2. Distribution of Pesticide Residues in Fresh Vegetables

A total of 300 vegetable samples were analysed for 218 pesticide residues, of which 93 (31%) contained at least one residue at a quantifiable concentration, while no quantifiable residues were detected in the remaining 207 samples (69%). Residue occurrence varied considerably among commodities, with detection frequencies of 49.3% in pepper, 34.7% in cucumber, 32% in tomato, and 8% in courgette. Pepper showed the highest residue detection frequency, whereas courgette showed the lowest, while similar frequencies were observed in cucumber and tomato. A total of 23 different pesticides were detected across the four vegetable groups, indicating marked commodity-specific variation in both residue occurrence and diversity. The concentrations of the detected pesticides are provided in Table S4.

Multiple residues were common among positive samples. Of the 93 samples containing quantifiable residues, 39 (41.9%) contained a single pesticide, and 54 (58.1%) contained two or more residues. Multiple residues were detected in 24 pepper, 16 cucumber, 13 tomato, and one courgette sample. Among pepper samples, 13 contained one residue, 17 contained two, six contained three, and one contained four pesticides. Among cucumber samples, 10 contained one residue, nine contained two, five contained three, one contained four, and one contained five pesticides. In tomato, 11 samples contained one residue, seven contained two, five contained three, and one contained four pesticides, whereas five courgette samples contained a single residue and one contained two residues. The maximum number of co-occurring residues was five, observed in a single cucumber sample, whereas pepper had the greatest number of samples containing multiple residues.

**Figure 3:**
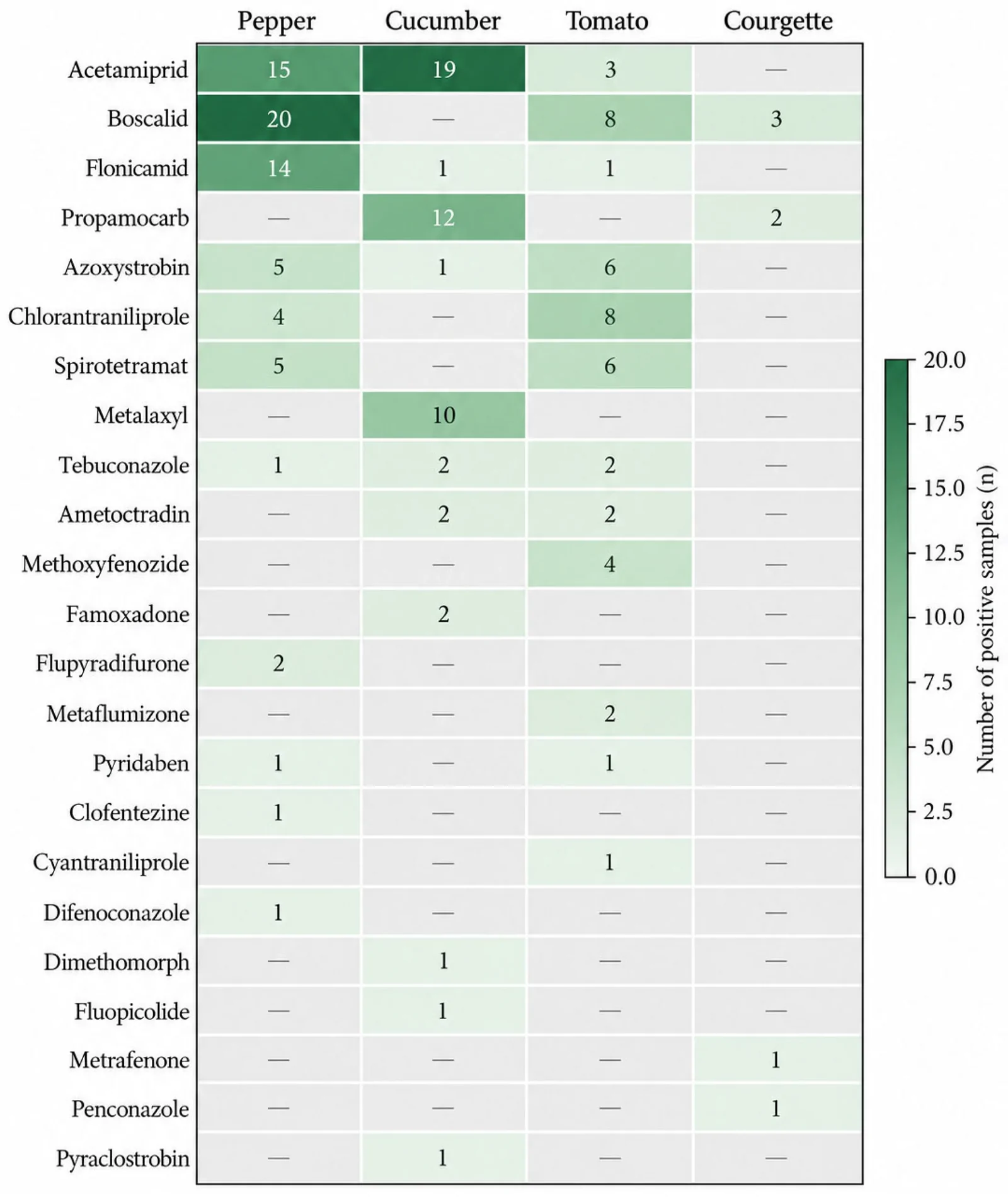
Distribution of pesticide residues detected in pepper, cucumber, tomato, and courgette samples. Cell values indicate the number of samples in which each pesticide was detected (*n*), with colour intensity reflecting the number of detections. Dashes indicate pesticides not detected in the respective commodity.

The diversity and frequency of detected pesticides also differed among commodities (Figure 3). Twelve different active substances were detected in tomato, 11 each in pepper and cucumber, and four in courgette. Boscalid (*n* = 20), acetamiprid (*n* = 15), and flonicamid (*n* = 14) were the most frequently detected pesticides in pepper, whereas acetamiprid (*n* = 19), propamocarb (*n* = 12), and metalaxyl (*n* = 10) predominated in cucumber. In tomato, boscalid and chlorantraniliprole were each detected in eight samples, followed by azoxystrobin and spirotetramat, each detected in six samples. Boscalid was the most frequently detected pesticide in courgette (*n* = 3). Concentrations of boscalid ranged from 0.016 to 0.128 mg kg^−1^ in pepper, 0.036 to 0.246 mg kg^−1^ in tomato, and 0.016 to 0.056 mg kg^−1^ in courgette, while acetamiprid concentrations in cucumber ranged from 0.033 to 0.741 mg kg^−1^. The highest pesticide concentration recorded in the study was 0.784 mg kg^−1^ for metalaxyl in cucumber. The maximum concentrations recorded in pepper and tomato were 0.385 and 0.639 mg kg^−1^, respectively, both for spirotetramat, while propamocarb showed the highest concentration in courgette (0.131 mg kg^−1^) (Table S4).

Assessment against the EU MRLs showed that 19 samples (6.3%) contained at least one residue exceeding the respective MRL. MRL exceedances were observed in 13 cucumber samples (17.3%), three pepper samples (4%), and three tomato samples (4%), whereas none were identified in courgette. Notably, cucumber had the highest proportion of MRL exceedances despite a lower residue detection frequency than pepper. Active substances not approved in the EU were also detected, including clofentezine and spirotetramat in pepper, dimethomorph and famoxadone in cucumber, and metaflumizone, methoxyfenozide, and spirotetramat in tomato. No unauthorised active substances were detected in courgette. These findings demonstrate that residue detection frequency alone does not adequately reflect regulatory compliance, highlighting the importance of considering both MRL exceedances and the authorisation status of detected active substances.

### 3.3. Comparison of Pesticide Residues in Fresh Vegetables with Previous Studies

Considerable variation in pesticide residue occurrence has been reported among vegetable monitoring studies, reflecting differences in geographical origin, agricultural practices, pesticide-use patterns, sampling strategies, and analytical scope (Table 1). For pepper, detection frequencies ranging from 27% to 100% have been reported internationally, while values of 48.1–100% have been documented in Türkiye [14,15,29,30]. The detection frequency observed in the present study (49.3%) was particularly close to the rates reported for jalapeño (48.1%) and sweet Italian-type pepper (52.6%) in Türkiye [15]. Higher detection frequencies have been reported in Bangladesh (95%) and Saudi Arabia (73.5%), whereas a lower frequency of 27% was reported in Serbia [30,31,32].

A different pattern was observed for cucumber. The detection frequency observed in the present study (34.7%) was lower than the 50–70% previously reported in Türkiye and below most values reported elsewhere [10,42]. Detection frequencies ranging from 52.8% to 100% have been reported in cucumber samples from China, Egypt, Iran, and Serbia [12,36,38,39,40,41]. Despite this comparatively low detection frequency, cucumber had the highest MRL exceedance rate (17.3%) among the vegetables examined in the present study. Previous investigations have similarly shown substantial variation in regulatory compliance, with MRL exceedance rates ranging from 0–3.3% in some studies to 29.7–40% in Egypt and China, while an exceedance rate of 100% was reported in one study from Iran. These comparisons further demonstrate that a higher residue detection frequency does not necessarily indicate a higher rate of MRL exceedance.

For tomato, the detection frequency of 32% was comparable to the 30% reported in Italy and the 37.5–40% reported in previous studies from Türkiye [10,42,46]. Higher detection frequencies have been reported in Albania (78.3%), Bangladesh (94.1%), and Burkina Faso (62.2%), as well as in some studies from Pakistan [43,44,45,48]. In courgette, only 8% of samples contained quantifiable residues. This frequency was lower than those reported in Kuwait (17%) and Serbia (13%) and substantially below the 75–100% reported in studies from Morocco and Jordan [34,36,49,50,51]. Despite the marked differences in detection frequency, the absence of MRL exceedances in courgette was consistent with findings reported from Cape Verde, Kuwait, Morocco, and Serbia [33,36,49,51].

The profiles of predominant pesticides also varied among studies, although some similarities were observed across commodities and geographical regions. Boscalid, which was frequently detected in pepper, tomato, and courgette in the present study, has also been reported among the predominant residues in peppers and tomatoes from Türkiye and in peppers from Serbia [14,15,30,42]. Acetamiprid was the second most frequently detected pesticide in pepper and the most frequently detected pesticide in cucumber. Acetamiprid has also been frequently reported in cucumber samples from Türkiye and among the predominant residues in peppers from Egypt, Saudi Arabia, and Bangladesh [13,31,32,42]. Other active substances, including procymidone, dimethomorph, imidacloprid, chlorfenapyr, and diazinon, have been reported as predominant residues in these vegetable groups in different regions [12,13,38,39,40,49]. The variability in predominant pesticide profiles is likely to reflect differences in authorised uses, pest and disease pressure, cultivation systems, and regional plant-protection practices.

### 3.4. Residue Quality Assessment Using the IqR

The *IqR* provides an integrated measure of residue quality by considering both the number of pesticide residues detected in a sample and their concentrations relative to the respective MRLs [18]. Unlike conventional MRL-based assessment, which evaluates individual pesticide residues separately, the *IqR* enables the overall residue profile of a sample to be characterised and may therefore identify samples of lower residue quality even when individual residues comply with their respective MRLs. However, the *IqR* classifications should not be interpreted as indicators of toxicological safety, as the index does not account for differences in pesticide toxicity or dietary exposure.

**Figure 4:**
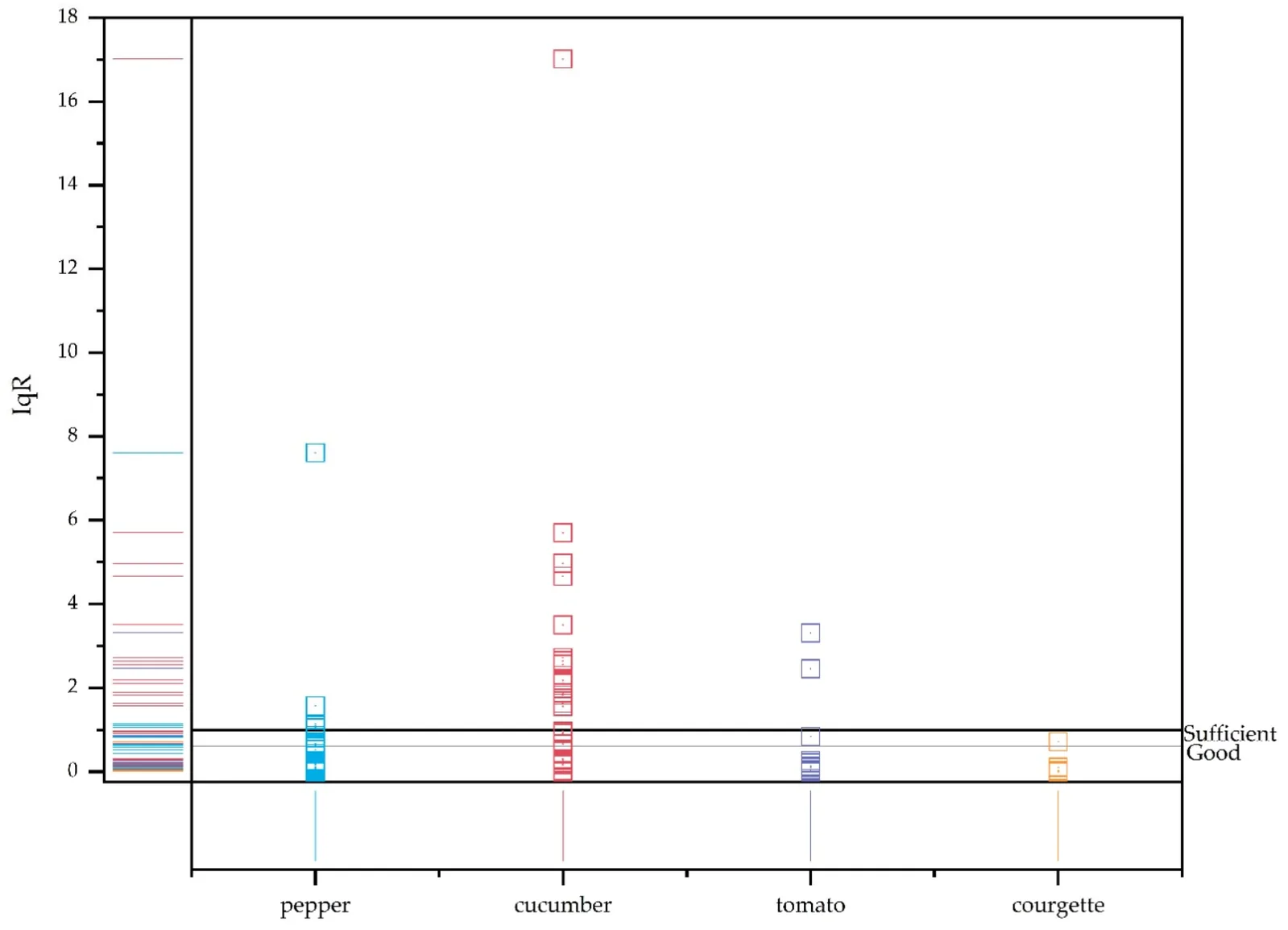
Distribution of pepper, cucumber, tomato, and courgette samples across quality categories based on the Index of Quality for Residues (*IqR*).

Marked differences in *IqR* classifications were observed among the four vegetable groups (Figure 4). Among pepper samples, 50.7% were classified as excellent (*IqR* = 0), 33.3% as good (0 < *IqR* ≤ 0.6), 6.7% as adequate (0.6 < *IqR* ≤ 1), and 9.3% as inadequate (*IqR* > 1). For cucumber, 65.3% of the samples were classified as excellent, 9.3% as good, 6.7% as adequate, and 18.7% as inadequate. A higher proportion of tomato samples was classified as excellent (68%), while 26.7% were classified as good, 1.3% as adequate, and 4% as inadequate. Courgette had the most favourable residue quality profile, with 92% of samples classified as excellent, 6.7% as good, and 1.3% as adequate, while none were classified as inadequate.

Further examination of samples classified as inadequate showed considerable variation in *IqR* values among commodities. Of the seven inadequate pepper samples, six had *IqR* values between 1 and 2, while one sample had a markedly higher value of 7.6. Among the 14 inadequate cucumber samples, four had values between 1 and 2, nine between 2 and 6, and one reached an *IqR* of 17, the highest value observed in the present study. The three inadequate tomato samples had *IqR* values between 2 and 3. No courgette samples had an *IqR* above 1. These findings indicate that most samples had favourable residue quality profiles, although markedly elevated *IqR* values were observed in individual samples, particularly in cucumber.

The proportion of pepper samples classified as inadequate (9.3%) was lower than the 27% reported for different pepper varieties grown under greenhouse conditions in Türkiye [14]. Previous studies have reported inadequate quality classifications for 15.8% of sweet Italian-type peppers and 5.7% of jalapeño peppers in Türkiye, while a considerably higher rate of 53.6% was reported for hot peppers in Saudi Arabia [15,52]. For cucumber, the inadequate quality rate observed in the present study (18.7%) was substantially lower than the 58.3% reported in Saudi Arabia [52]. In China, *IqR* values below 0.6 were reported for all open-field cucumber samples and 89.4% of greenhouse cucumber samples [12]. For tomato, the inadequate quality rate observed in the present study (4%) was considerably lower than the 26.9% reported in Saudi Arabia [52]. In a study conducted in Uganda, most market vegetables were also classified as having good residue quality, although samples classified as inadequate were identified [53]. Differences in commodity type and variety, sampling design, analytical scope, and the MRLs applied may limit direct comparisons of *IqR* classifications across studies.

### 3.5. Risk-Based Ranking of Pesticide Residues

**Figure 5:**
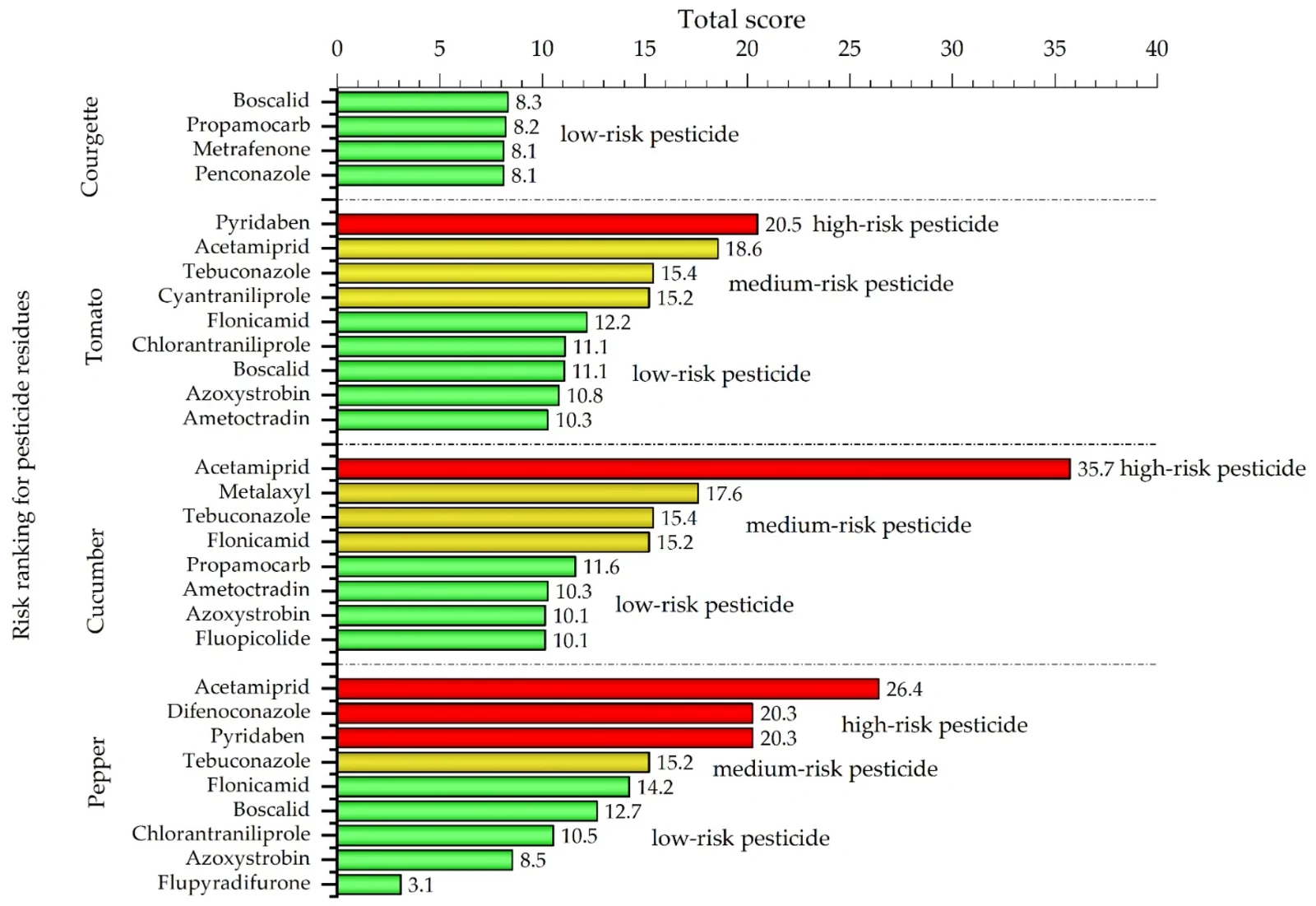
Risk-based ranking of pesticide residues detected in pepper, cucumber, tomato, and courgette based on composite risk scores. Pesticides were classified into high (≥20), medium (15–<20), and low (<15) risk-ranking categories.

The risk-based ranking of the 23 pesticide residues detected in the vegetable samples is presented in Figure 5. Based on the composite risk scores, pesticides were classified into high (≥20), medium (15–<20), and low (<15) ranking categories. Acetamiprid, difenoconazole, and pyridaben were assigned to the high category in at least one vegetable group. The highest score was obtained for acetamiprid in cucumber (35.7), followed by acetamiprid in pepper (26.4). Pesticides assigned to the high category were identified in pepper, cucumber, and tomato, whereas none of those evaluated in courgette reached the high-category threshold.

In pepper, acetamiprid had the highest risk score (26.4), followed by difenoconazole and pyridaben, each of which was assigned to the high category with a score of 20.3. Tebuconazole was classified in the medium category (15.2), whereas the remaining pesticides had scores ranging from 3.1 to 14.2 and were classified as low. In cucumber, acetamiprid had the highest score (35.7) and was the only pesticide assigned to the high category. Metalaxyl (17.6), tebuconazole (15.4), and flonicamid (15.2) were classified as medium, while the remaining pesticides were classified as low.

In tomato, pyridaben had the highest score (20.5) and was the only pesticide assigned to the high category. Acetamiprid (18.6), tebuconazole (15.4), and cyantraniliprole (15.2) were classified as medium, while the remaining pesticides were classified as low. All four pesticides evaluated in courgette were assigned to the low category, with scores ranging from 8.1 to 8.3. The ranking therefore identified acetamiprid as the highest-priority pesticide overall, particularly in cucumber and pepper, while pyridaben was assigned to the high category in both pepper and tomato, and difenoconazole in pepper.

A previous risk-ranking assessment of 31 pesticides detected in 197 pepper samples from Serbia classified methiocarb, formetanate, chlorpyrifos, pirimiphos-methyl, flutriafol, azinphos-methyl, and difenoconazole in the high category [30]. Although the pesticides assigned to the high category differed between the two studies, difenoconazole was classified in this category in both. Differences in the pesticides prioritised by risk-ranking approaches may reflect variation in residue occurrence and concentrations, toxicological reference values, pesticide-use patterns, and the scoring criteria applied. The rankings obtained in the present study should therefore be interpreted within the assumptions of the adopted scoring framework, particularly the uniform assignment of index E, as their robustness to alternative scoring assumptions was not evaluated.

### 3.6. Deterministic Dietary Exposure and Risk Characterisation

For chronic dietary exposure, the UB estimates from pepper consumption were 0.0002 and 0.0003 mg kg^−1^ bw day^−1^ for adults and children, respectively. The respective estimates were 0.0001 and 0.0001 mg kg^−1^ bw day^−1^ for cucumber, 0.0007 and 0.0009 mg kg^−1^ bw day^−1^ for tomato, and 9.0 × 10^−6^ and 1.1 × 10^−5^ mg kg^−1^ bw day^−1^ for courgette. Among the four vegetables, the highest total exposure was estimated for tomato and the lowest for courgette, indicating that residue occurrence alone did not determine the magnitude of dietary exposure.

The *HQc* values estimated for individual pesticides are presented in Table S5. For individual pesticides, *HQc* values remained below 1 for both consumer groups under all three exposure scenarios. In pepper, *HQc* values ranged from 2.8 × 10^−6^ to 0.0033 (LB) and from 1.0 × 10^−5^ to 0.0053 (UB) for adults, compared with 3.4 × 10^−6^–0.0040 (LB) and 1.3 × 10^−5^–0.0065 (UB) for children. In cucumber, *HQc* values ranged from 2.3 × 10^−7^ to 0.0044 (LB) and from 8.1 × 10^−7^ to 0.0053 (UB) for adults, compared with 2.7 × 10^−7^–0.0054 (LB) and 9.9 × 10^−7^–0.0065 (UB) for children. In tomato, the ranges were 4.3 × 10^−7^–0.0064 (LB) and 5.2 × 10^−6^–0.0157 (UB) for adults, and 5.2 × 10^−7^–0.0078 (LB) and 6.3 × 10^−6^–0.0192 (UB) for children. The lowest *HQc* values were observed for courgette, ranging from 5.5 × 10^−7^ to 7.9 × 10^−6^ (LB) and from 8.4 × 10^−6^ to 6.9 × 10^−5^ (UB) for adults, and from 6.7 × 10^−7^ to 9.6 × 10^−6^ (LB) and from 1.0 × 10^−5^ to 8.5 × 10^−5^ (UB) for children.

**Figure 6:**
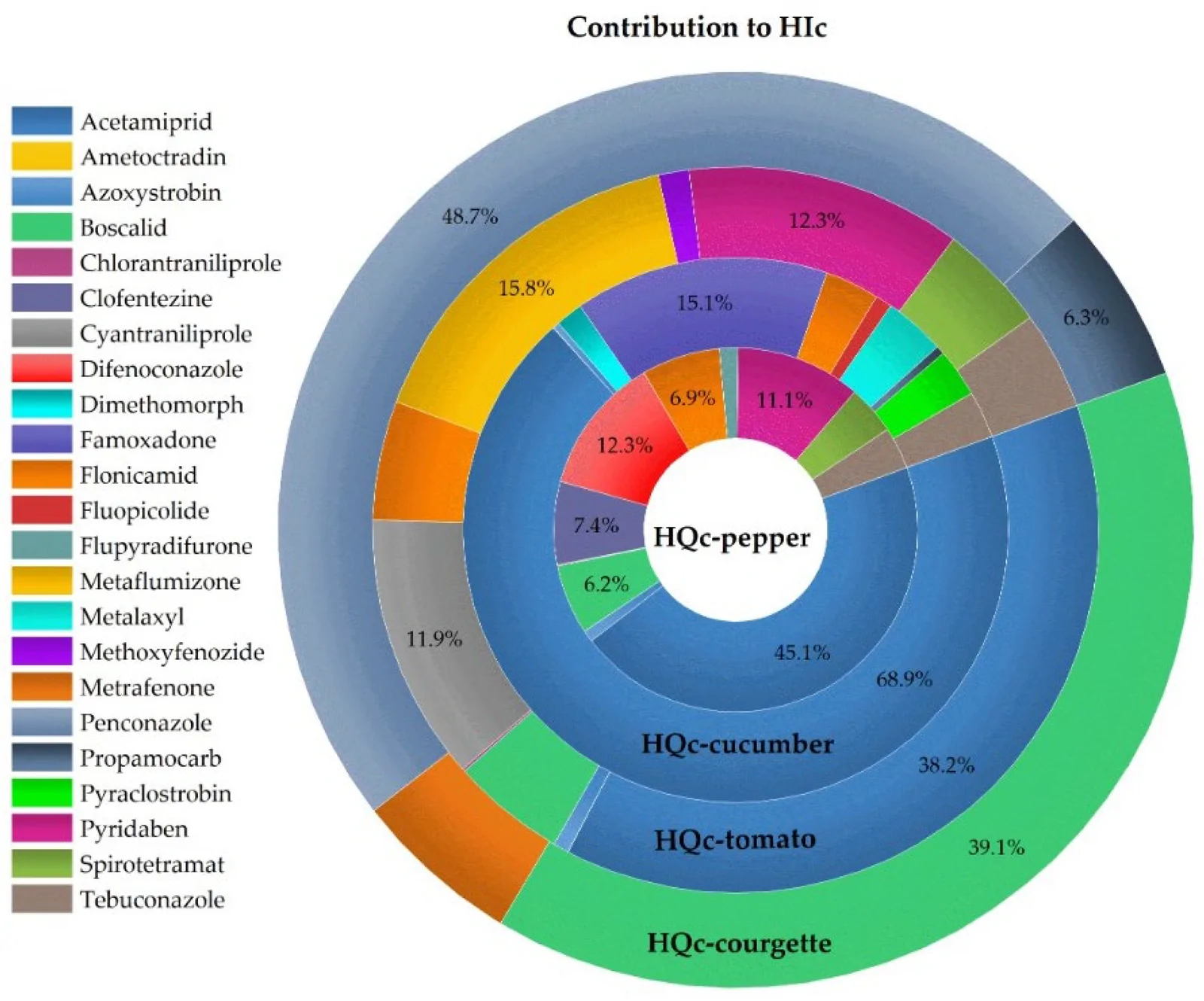
Relative contributions of individual pesticides to the commodity-specific chronic hazard index (*HIc*) for pepper, cucumber, tomato, and courgette in adults and children.

At the commodity level, *HIc* values were 0.0118 and 0.0144 for pepper, 0.0077 and 0.0094 for cucumber, 0.0394 and 0.0502 for tomato, and 0.0001 and 0.0002 for courgette in adults and children, respectively. Tomato had the highest commodity-specific *HIc*, whereas the lowest values were obtained for courgette. The relative contributions of individual pesticides to the commodity-specific *HIc* values are shown in Figure 6. Acetamiprid was the principal contributor to *HIc* in pepper (45.1%), cucumber (68.9%), and tomato (38.2%) for both consumer groups, whereas penconazole was the principal contributor in courgette (48.7%). For the combined consumption of all four vegetables, the summed *HIc* was 0.0591 for adults and 0.0741 for children under the UB scenario.

All individual *HQc* values were below 1, indicating that chronic exposure to each pesticide considered separately remained below its toxicological reference value under the scenarios evaluated. The summed *HIc* values also remained well below 1 for each commodity and for the combined consumption of all four vegetables. These findings suggest a low level of chronic dietary concern for the pesticide residues evaluated in both adults and children. However, because *HIc* was derived by summing *HQc* values across pesticides irrespective of their specific toxicological modes of action, it should be interpreted as a conservative screening-level indicator rather than evidence of toxicological dose additivity among all detected pesticides.

**Figure 7:**
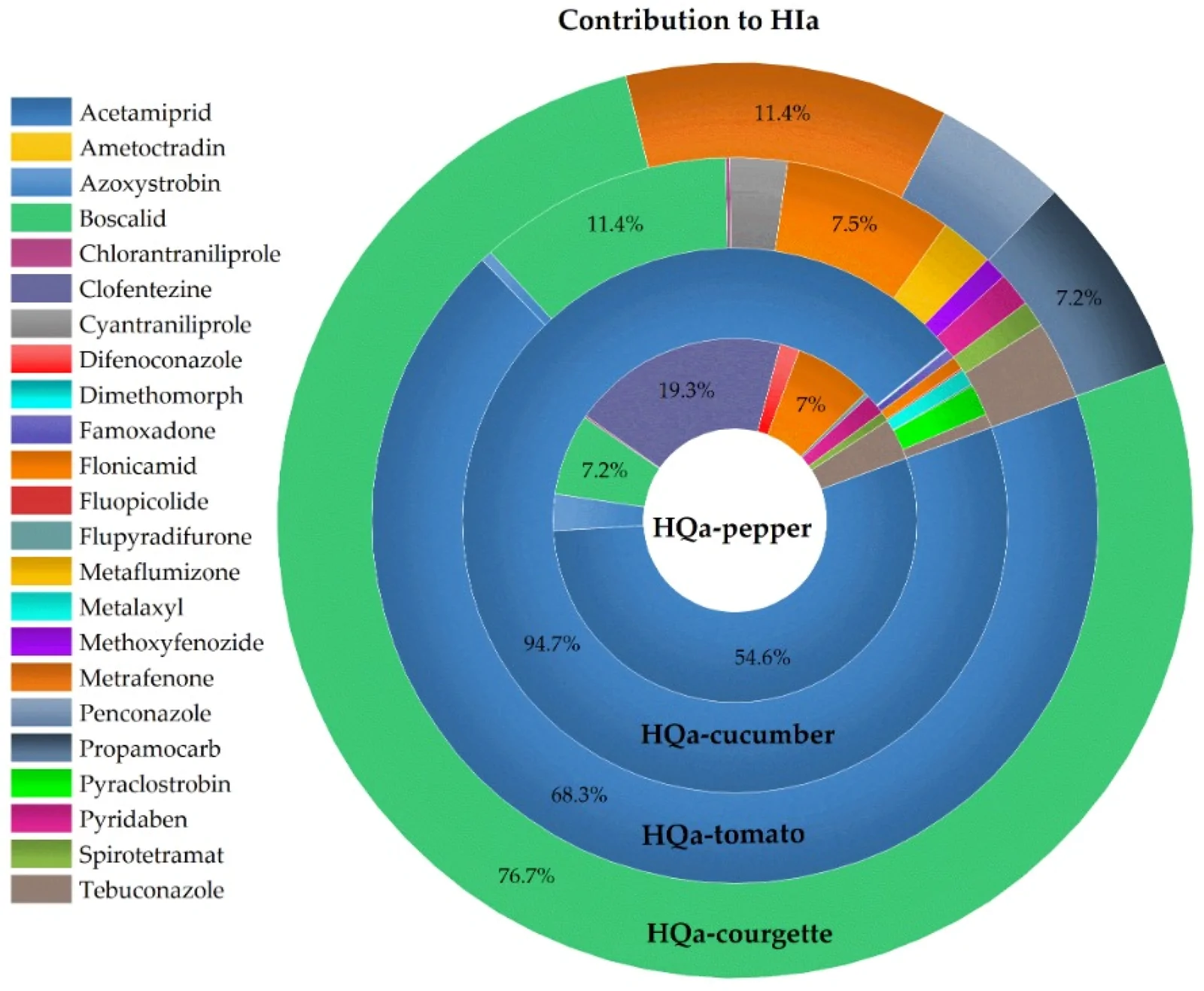
Relative contributions of individual pesticides to the commodity-specific acute hazard index (*HIa*) for pepper, cucumber, tomato, and courgette in adults and children.

Acute dietary exposure showed a different pattern. *HQa* values calculated using the highest measured concentrations of individual pesticides are presented in Table S5. In pepper, *HQa* values ranged from 0.0009 to 0.2822 for adults and from 0.0025 to 0.7642 for children. In cucumber, *HQa* values ranged from 0.0006 to 3.7465 for adults and from 0.0010 to 5.8447 for children. In tomato, the ranges were 0.0001–0.5048 for adults and 0.0002–1.4073 for children. In courgette, *HQa* values ranged from 0.0003 to 0.0044 for adults and from 0.0003 to 0.0048 for children. The relative contributions of individual pesticides to the commodity-specific *HIa* values are shown in Figure 7. Acetamiprid was the principal contributor to *HIa* in pepper (54.6%), cucumber (94.7%), and tomato (68.3%), whereas boscalid was the principal contributor in courgette (76.7%).

Acetamiprid exceeded the acute risk threshold (*HQa* > 1) in three cucumber samples for adults and 11 cucumber samples for children. In tomato, *HQa* exceeded 1 in two samples, both for children, whereas no threshold exceedances were identified for pepper or courgette. In contrast to the consistently low chronic risk estimates, potential acute dietary concern was therefore identified for individual cucumber and tomato samples, particularly for children.

The low chronic risk estimates obtained in the present study are broadly consistent with previous findings for pepper. Boudriiya et al. [35] reported deterministic *HIc* values of 0.044 and 0.046 for adults and children/adolescents, respectively, following green pepper consumption. A deterministic and probabilistic assessment of sweet Italian-type and jalapeño peppers in Türkiye similarly showed that chronic dietary risk remained well below the relevant safety thresholds for adults, with lambda-cyhalothrin and acetamiprid identified as the principal contributors to chronic exposure [15]. Lučić and Onjia [30] also reported acceptable chronic risk estimates for pepper consumption in Serbia across all consumer groups evaluated, including children and pregnant women.

For cucumber, the low chronic risk estimated in the present study is consistent with some previous findings, although substantially higher estimates have also been reported. Mahdavi et al. [11] calculated *HIc* values of 0.067 and 0.30 for adults and children, respectively, from 100 greenhouse-grown cucumber samples in Iran, with both values remaining below 1. Khoshnam et al. [40], however, reported *HIc* values of 0.23 for adults and 1.06 for children following cucumber consumption, together with values of 0.33 and 1.51, respectively, for tomato. Chronic and acute dietary risks associated with open-field and greenhouse-grown cucumber consumption in China were also reported to remain at acceptable levels across all consumer groups [12]. Variation among studies may reflect differences in residue concentrations and profiles, food consumption amounts, body weight, consumer groups, toxicological reference values, and methodological assumptions used in exposure estimation.

Although pepper had the highest residue detection frequency in the present study, tomato produced the highest commodity-specific *HIc* values. This finding further demonstrates that residue occurrence alone is not sufficient to characterise dietary risk, which is also influenced by residue concentrations, consumption levels, and pesticide-specific toxicological reference values. In a four-year monitoring study of tomato samples in Italy, chronic exposure ranged from 0 to 36% of the ADI for adults and the general population and from 0 to 10% for children, toddlers, and infants, with no individual pesticide exceeding its ADI [46]. In contrast, the *HIc* values above 1 reported for children consuming tomato in Iran illustrate how differences in residue profiles and exposure assumptions can produce markedly different risk estimates for the same commodity [40]. In the present study, the summed *HIc* remained well below 1 even under the worst-case scenario incorporating consumption of all four vegetables.

Although chronic dietary concern was low, the *HQa* exceedances identified for acetamiprid in cucumber and tomato demonstrate that low chronic risk estimates do not necessarily exclude potential short-term concern associated with individual samples containing comparatively high residue concentrations. A similar distinction between chronic and acute risk has been reported for cucumber in Egypt, where chronic risk remained below the relevant thresholds, whereas acute exposure to carbosulfan, thiophanate-methyl, and acetamiprid was reported to exceed the ARfD under some scenarios, with the highest estimate reaching 395% of the ARfD for carbosulfan [39]. Lučić and Onjia [30] reported acceptable chronic risk estimates for pepper across consumer groups, while acute risk indices exceeded 1 in some individual samples, particularly for children. For sweet Italian-type and jalapeño peppers in Türkiye, acute risk was generally low, although a potential acute concern associated with lambda-cyhalothrin was identified for jalapeño pepper under high-exposure conditions [15].

Acetamiprid was particularly relevant to the acute risk profile in the present study. It produced the highest *HQa* values in pepper, cucumber, and tomato and was responsible for all *HQa* values exceeding 1 in cucumber and tomato. In the Italian tomato study of Atzei et al. [46], ARfD utilisation under the standard acute assessment ranged from 0.1% to 45.9% for adults and from 0.2% to 55.8% for toddlers. When the updated ARfD of 0.005 mg kg^−1^ bw day^−1^ for acetamiprid was applied, estimated exposure under the child consumption scenario reached 125.4% of the ARfD. This finding is consistent with the present study, in which the acute threshold for tomato was exceeded only in children and exclusively for acetamiprid. The identification of acetamiprid among the pesticides associated with potential acute concern in cucumber samples from Egypt further supports its relevance to short-term dietary exposure across different commodities and geographical settings [39].

Differences between adults and children were consistently observed in both chronic and acute assessments, with higher *HQc*, *HIc*, and *HQa* values generally estimated for children. This pattern primarily reflects the greater food intake relative to body weight in children under the exposure scenarios applied. Similar age-related differences have been reported in previous dietary risk assessments of pesticide residues in cucumber, tomato, and pepper [11,30,40]. In the present study, the larger number of cucumber samples with *HQa* values above 1 in children than in adults, together with threshold exceedances for tomato occurring exclusively in children, reinforces the importance of age-specific exposure assessment when evaluating dietary risks from pesticide residues.

### 3.7. Probabilistic Dietary Exposure and Risk Characterisation

**Figure 8:**
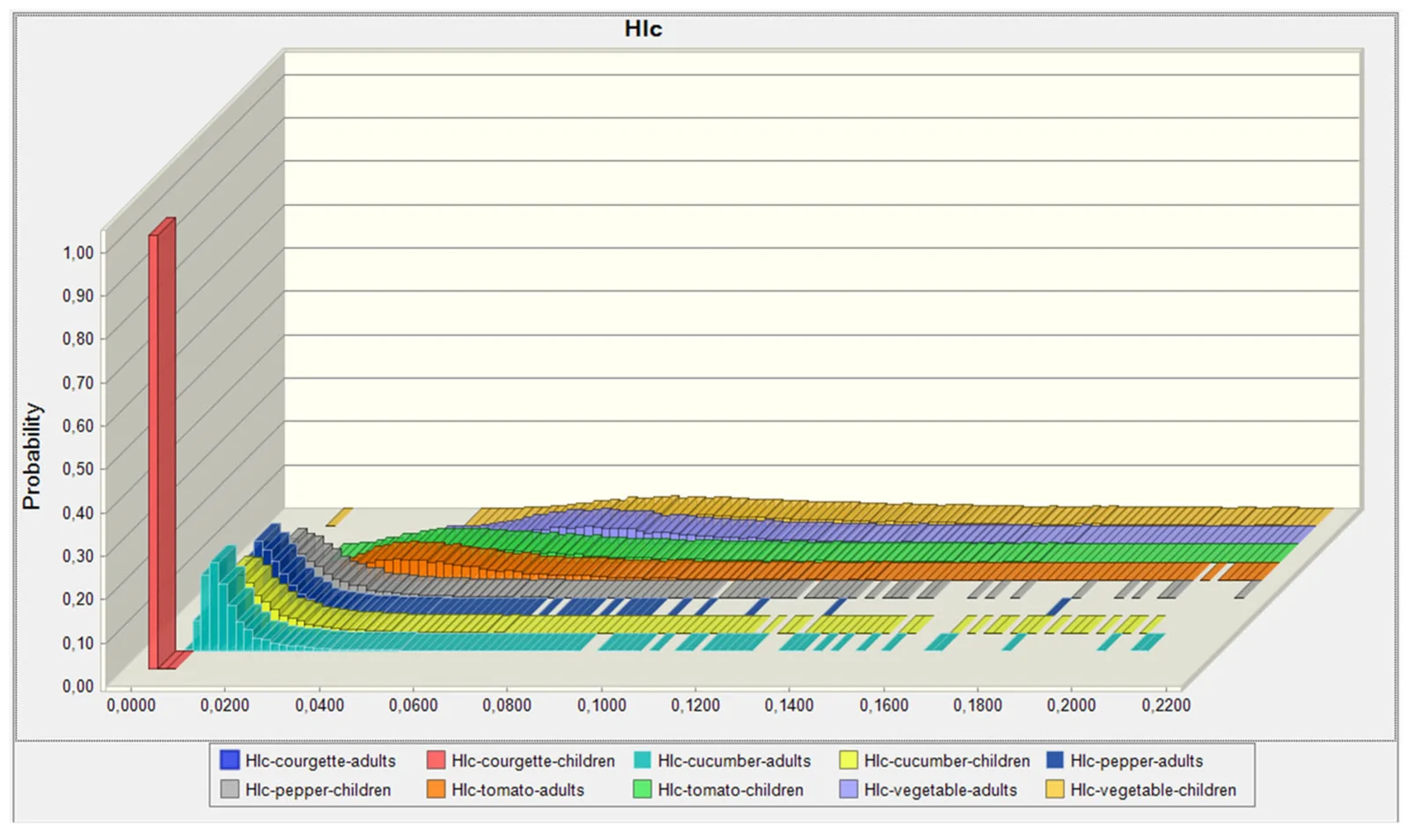
Probability distributions of the chronic hazard index (*HIc*) for pesticide residues in pepper, cucumber, tomato, and courgette estimated for adults and children using Monte Carlo simulation.

To complement the deterministic assessment and characterise the variability in chronic dietary exposure, a probabilistic approach based on MCS was applied. The resulting *HIc* distributions for adults and children following consumption of pepper, cucumber, tomato, and courgette are presented in Figure 8.

The simulated *HIc* distributions were right-skewed for both consumer groups across all four vegetables, with upper tails extending towards higher values. For pepper, the mean and median *HIc* values were 0.0168 and 0.0147 for adults and 0.0227 and 0.0181 for children, respectively. The P5-P95 ranges were 0.0093–0.0313 in adults and 0.0103–0.0462 in children. For cucumber, mean *HIc* values were 0.0148 for adults and 0.0207 for children, with median values of 0.0115 and 0.0144, respectively. The P5-P95 ranges were 0.0070–0.0315 for adults and 0.0079–0.0462 for children. In both vegetables, mean values exceeded the medians, reflecting the influence of higher values in the upper tails of the distributions.

The highest probabilistic chronic risk estimates among the four vegetables were obtained for tomato. The mean and median *HIc* values were 0.0523 and 0.0432 for adults and 0.0924 and 0.0543 for children, respectively, while the P5-P95 intervals were 0.0248–0.1075 and 0.0280–0.1581. Substantially lower values were obtained for courgette. At the graphical precision shown, the P5, median, mean, and P95 values were approximately 0.0001 for adults, while the P5 was 0.0001, the median and mean were 0.0002, and the P95 was 0.0004 for children. Across commodities, children generally showed higher central estimates and upper-percentile values than adults.

**Figure 9:**
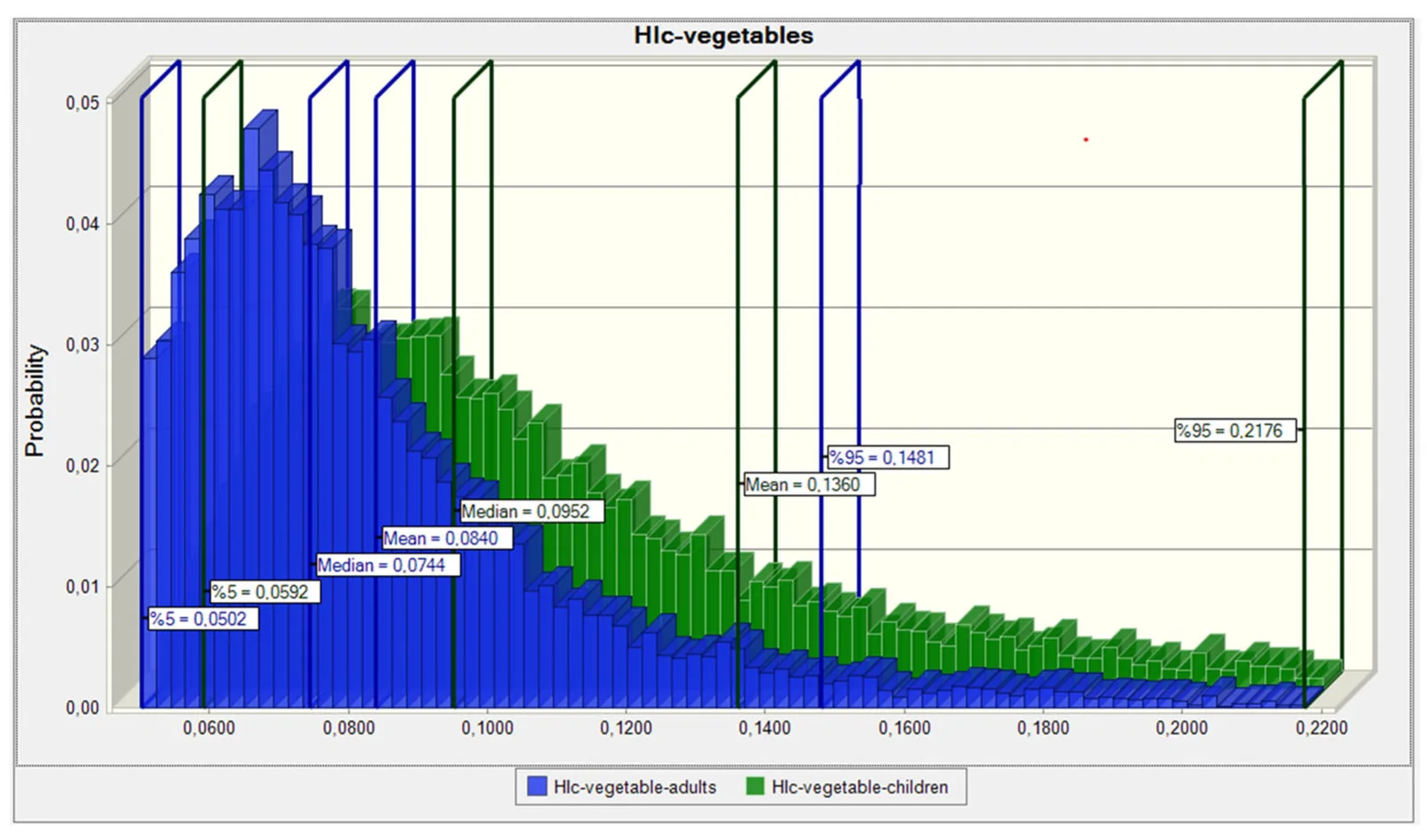
Probability distributions of the chronic hazard index (*HIc*) for pesticide residues associated with the combined consumption of pepper, cucumber, tomato, and courgette estimated for adults and children using Monte Carlo simulation.

Chronic risk associated with the combined consumption of all four vegetables was also evaluated, and the resulting *HIc* distributions are presented in Figure 9. The distributions were right-skewed in both consumer groups. For adults, the mean and median *HIc* values were 0.0840 and 0.0744, with P5 and P95 values of 0.0502 and 0.1481, respectively. For children, the mean increased to 0.1360 and the median to 0.0952, while the P5 and P95 values were 0.0592 and 0.2176. The greater separation between the mean and median in children, together with the higher P95, indicates a stronger influence of the upper tail on the simulated risk distribution.

**Figure 10:**
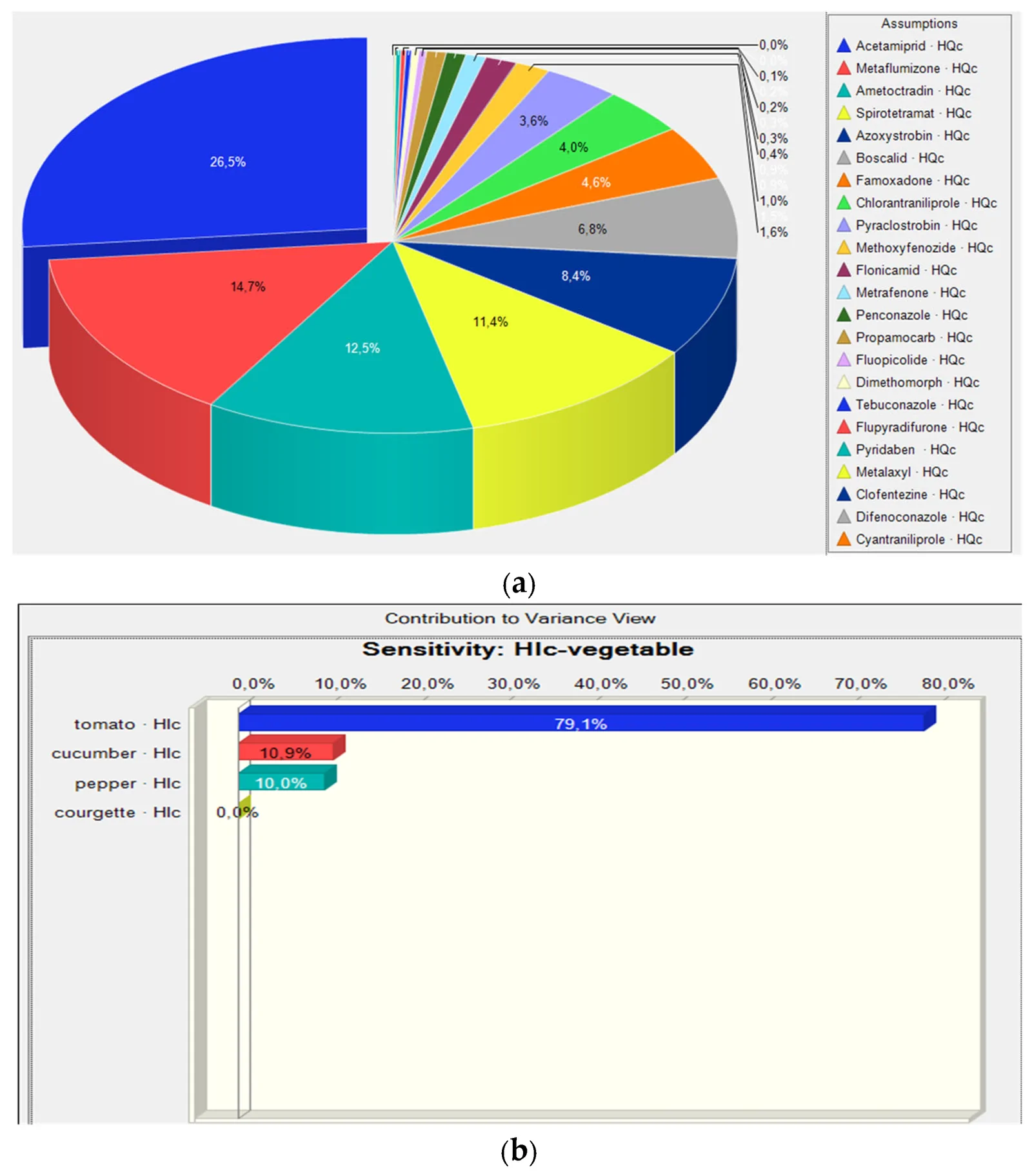
Sensitivity analysis of the probabilistic chronic dietary risk model based on contributions to variance in the *HIc* for the combined consumption of all four vegetables: (**a**) individual pesticides and (**b**) vegetable commodities.

Sensitivity analysis was performed to identify the principal contributors to variability in the combined-consumption *HIc* (Figure 10). At the commodity level, tomato accounted for 79.1% of the total variance, substantially exceeding the contributions of cucumber (10.9%) and pepper (10.0%), while the contribution of courgette was negligible. At the pesticide level, acetamiprid was the largest contributor to variance (26.5%), followed by metaflumizone (14.7%), ametoctradin (12.5%), spirotetramat (11.4%), azoxystrobin (8.4%), and boscalid (6.8%). Famoxadone, chlorantraniliprole, and pyraclostrobin made smaller contributions of 4.6%, 4.0%, and 3.6%, respectively, while the contribution of each remaining pesticide was below 2%. The predominance of tomato at the commodity level is consistent with the higher probabilistic *HIc* estimates obtained for this vegetable and indicates that tomato-related inputs were the principal source of variability in the combined chronic risk distribution. At the pesticide level, variance was distributed across several active substances rather than being dominated by a single pesticide, although acetamiprid exerted the greatest individual influence.

All probabilistic *HIc* estimates remained below 1 for both individual vegetables and their combined consumption. At the P95 level, the highest *HIc* was 0.2176 for children under the combined-consumption scenario. The probabilistic assessment therefore indicates a low level of chronic dietary concern for the pesticide residues evaluated, including at the upper end of the modelled exposure distributions. As in the deterministic assessment, these summed *HIc* values should be interpreted as screening-level estimates because *HQc* values were aggregated across pesticides without consideration of common mechanisms or modes of toxicological action.

The low probabilistic chronic risk estimates are broadly consistent with previous assessments of pesticide residues in pepper. In Türkiye, probabilistic assessment of sweet Italian-type and jalapeño peppers indicated that chronic dietary risk remained well below the relevant safety threshold for adults, with lambda-cyhalothrin and acetamiprid among the principal contributors [15]. Boudriiya et al. [35] also reported low cumulative risk estimates for green pepper consumption in Morocco, with upper-percentile values ranging from 0.0006 to 0.0045 in adults and from 0.0011 to 0.0057 in children/adolescents. For cucumber, a wider range of probabilistic estimates has been reported. Mahdavi et al. [11] reported *HIc* values of 0.067 for adults and 0.30 for children following consumption of greenhouse-grown cucumber in Iran, whereas Yousefi et al. [41] reported *HIc* values of 2.22 and 1.36 for children consuming unwashed and water-washed cucumbers, respectively, in an assessment focused on organophosphate pesticides. Differences among studies may reflect variation in residue profiles and concentrations, consumption data, body-weight assumptions, toxicological reference values, treatment of censored data, and probabilistic modelling approaches.

The probabilistic analysis extended the deterministic assessment by describing the distribution of chronic risk rather than relying solely on point estimates. This was particularly relevant for children, for whom both central and upper-percentile *HIc* values were consistently higher than those estimated for adults. The combined-consumption model further allowed the contribution of all four vegetables to be incorporated within a single simulated distribution. Evaluation of the upper tails therefore provided a more informative characterisation of high-exposure scenarios than deterministic estimates alone. However, the use of national food balance-sheet data rather than individual dietary consumption records introduces uncertainty into exposure estimates, particularly regarding differences in consumption patterns among population groups.

## 4. Conclusions

The findings of this study highlight commodity-specific differences in pesticide residue occurrence, regulatory compliance, residue quality, and dietary risk among pepper, cucumber, tomato, and courgette sampled from supermarkets in Alanya, Antalya and Corum, Türkiye. The higher detection frequency observed in pepper, contrasted with the greater frequency of MRL exceedances in cucumber, indicates that monitoring priorities should not be based solely on the occurrence of pesticide residues. The *IqR* assessment provided an additional measure of residue quality, while risk ranking identified acetamiprid as the highest-priority pesticide, particularly in cucumber and pepper. Under the evaluated exposure scenarios, deterministic and probabilistic assessments indicated a low level of chronic dietary concern for both adults and children. In contrast, potential acute concern was identified for acetamiprid in individual cucumber and tomato samples, particularly among children, demonstrating the importance of considering short-term exposure and vulnerable consumer groups separately from chronic exposure. The probabilistic assessment supported the chronic findings, with *HIc* values remaining below 1 for individual commodities and their combined consumption, including at the upper end of the simulated distributions. As exposure estimates were based on residue concentrations in unprocessed commodities, potential changes associated with household washing, peeling, or cooking were not considered and may influence actual dietary exposure. Because the summed *HIc* values were derived by aggregating *HQc* values across pesticides irrespective of their toxicological modes of action, these estimates should be interpreted as conservative screening-level indicators rather than evidence of dose additivity. The integration of residue occurrence, regulatory compliance, residue quality, risk-based prioritisation, and deterministic and probabilistic dietary assessment provides a more informative basis for pesticide residue surveillance than reliance on detection frequency or MRL compliance alone. Particular attention to commodities and pesticides associated with MRL exceedances or potential acute dietary concern may support more targeted, risk-based monitoring strategies.

## Figures and Tables

**Table 1:** Comparison of pesticide residue occurrence in fresh vegetables across studies.

Vegetable	Country	No. of Samples (*n*)	Detection Frequency (%)	Samples >MRL (%)	Most Frequently Detected Pesticides	Reference
Pepper	Bangladesh	60	95	0	Thiamethoxam, acetamiprid	[31]
Pepper	Cape Verde	34	NR ^a^	0	Deltamethrin	[33]
Pepper	Egypt	208	66.4	2.9	Acetamiprid	[13]
Pepper	Jordan	15	53.3	NR	Imidacloprid	[34]
Pepper	Morocco	80	37.5	3.8	Imidacloprid, pirimicarb	[35]
Pepper	Saudi Arabia	536	73.5	23.5	Imidacloprid, acetamiprid	[32]
Pepper	Serbia	197	27	4.1	Boscalid, azoxystrobin	[30]
Pepper	Serbia	124	46	1.6	NR	[36]
Pepper	Tanzania	5	100	NR	NR	[37]
Pepper	Türkiye	48	100	NR	Pyridaphention, dinoseb, diazinon	[29]
Pepper	Türkiye	10	60	10	Pyridaben, acetamiprid	[10]
Pepper	Türkiye	100	98	20	Boscalid, fluopyram	[14]
Pepper (jalapeño)	Türkiye	106	48.1	5.7	Boscalid	[15]
Pepper (sweet Italian-type)	Türkiye	76	52.6	15.8	Flonicamid	[15]
Pepper	Türkiye	75	49.3	4	Boscalid, acetamiprid, flonicamid	Present study
Cucumber	Cape Verde	24	NR	0	Deltamethrin, dimethoate	[33]
Cucumber	China	180	64.4	0	Dimethomorph	[12]
Cucumber	China	180	85.0	2.8	Boscalid	[12]
Cucumber	China	60	95	40	Procymidone	[38]
Cucumber	Egypt	387	65.4	29.7	Chlorfenapyr, metalaxyl, fluopyram	[39]
Cucumber	Iran	15	93	0	Diazinon	[40]
Cucumber	Iran	24	100	100	Dimethoate, diazinon	[41]
Cucumber	Iran	100	NR	18	Imidacloprid	[11]
Cucumber	Serbia	91	52.8	3.3	NR	[36]
Cucumber	Tanzania	5	100	NR	NR	[37]
Cucumber	Türkiye	10	70	20	Azoxystrobin, fluopyram	[10]
Cucumber	Türkiye	20	50	20	Acetamiprid	[42]
Cucumber	Türkiye	75	34.7	17.3	Acetamiprid	Present study
Tomato	Albania	23	78.3	8.7	Propamocarb, difenoconazole	[43]
Tomato	Bangladesh	68	94.1	0	Azoxystrobin, difenoconazole	[44]
Tomato	Burkina Faso	328	62.2	61.3	DDT, acetamiprid, lambda-cyhalothrin	[45]
Tomato	Cape Verde	134	NR	0	Dithiocarbamates	[33]
Tomato	Egypt	262	50	4.9	Chlorfenapyr	[13]
Tomato	Italy	360	30	0	NR	[46]
Tomato	Pakistan	113	22	1.8	Azoxystrobin, tebuconazole	[47]
Tomato	Pakistan	51	58.8	7.8	Imidacloprid, tebuconazole	[48]
Tomato	Serbia	187	46.5	1.1	NR	[36]
Tomato	Tanzania	5	100	NR	NR	[37]
Tomato	Türkiye	8	37.5	0	Pyridaben	[10]
Tomato	Türkiye	20	40	5	Boscalid, pyraclostrobin	[42]
Tomato	Türkiye	75	32	4	Boscalid, chlorantraniliprole	Present study
Courgette	Cape Verde	14	NR	0	Deltamethrin	[43]
Courgette	Kuwait	12	17	0	Imidacloprid	[49]
Courgette	Jordan	10	100	100	Chlorothalonil, demeton-S-methyl, sulfoxide, dichlofluanid, epoxiconazole, flamprop-methyl, iprodione	[50]
Courgette	Jordan	15	80	NR	Imidacloprid	[34]
Courgette	Morocco	4	75	0	NR	[51]
Courgette	Serbia	23	13	0	NR	[36]
Courgette	Türkiye	75	8	0	Boscalid	Present study

^a^ NR: Not reported.

## Data Availability

The original contributions presented in the study are included in the article and Supplementary Material; further inquiries can be directed to the corresponding author.

## Supplementary Materials

The following supporting information can be downloaded at: https://www.mdpi.com/article/10.3390/foods15213898/s1, Table S1: MS/MS parameters (precursor ions, product ions, and collision energies) for the target pesticides analysed by LC-HRMS/MS; Table S2: Definitions and scoring criteria for the indices used in pesticide residue risk ranking; Table S3: Scores assigned to indices A–F for the pesticide residue risk-ranking system; Table S4: Occurrence and concentration of pesticide residues detected in pepper, cucumber, tomato, and courgette samples; Table S5: Chronic and acute hazard quotients (*HQc* and *HQa*) for pesticide residues detected in pepper, cucumber, tomato, and courgette samples for adults and children.
