# Supplementary material for: Pesticide Residues in Fresh Vegetables in Türkiye: Regulatory Compliance, Risk Ranking, and Deterministic and Probabilistic Dietary Risk Assessment
Source: Foods. 2026 Oct 30;15(21):3898. doi: 10.3390/foods15213898 (PMC13763161; doi:10.3390/foods15213898)
Supplement: Supplementary file 1 [file foods-15-03898-s001.zip › foods-4609095-supplementary.pdf]

Supplementary Material

Pesticide Residues in Fresh Vegetables in Türkiye: Regulatory Compliance, Risk Ranking, and Deterministic and Probabilistic Dietary Risk Assessment

Sebahat Oztekin [1], Eylem Odabas [2], Ozgur Golge [3], Miguel Ángel González-Curbelo [4] and Bulent Kabak [1,2,*]

[1] Department of Food Engineering, Faculty of Agriculture, Ataturk University, Erzurum, 25240, Türkiye
[2] Department of Food Engineering, Faculty of Engineering and Natural Sciences, Hitit University, Corum, 19030, Türkiye
[3] Department of Gastronomy and Culinary Arts, Faculty of Tourism, Alanya Alaaddin Keykubat University, Alanya, 07425, Türkiye
[4] Departamento de Ciencias Básicas, Facultad de Ingeniería, Universidad EAN, Calle 79 n11-45, Bogotá D.C. 110221, Colombia
* Correspondence: bulentkabak@hitit.edu.tr; bulentkabak@grv.atauni.edu.tr (B.K.)

Table S1. MS/MS parameters (precursor ions, product ions, and collision energies) for the target pesticides analysed by LC-HRMS/MS.

Pesticide	Precursor ion (*m/z*)	Product ion (*m/z*)	CE* (eV)
Abamectin	890.4	305.1	-31
	890.4	145.2	-44
Acephate	184.1	125.1	18
	184.1	143.0	8
Acetamiprid	223.0	56.1	15
	223.0	126.0	20
Aldicarb	208.2	89.1	19
	208.2	116.1	8
Aldicarb sulfone	223.0	86.0	14
	223.0	148.0	10
Aldicarb sulfoxide	207.0	89.0	14
	207.0	132.0	10
Ametoctradin	276.3	149.1	39
	276.3	176.2	42
Amitraz	294.4	163.2	14
	294.4	253.3	12
Atrazine	216.1	96.1	23
	216.1	174.1	18
Atrazine-desisopropyl	174.1	104.1	33
	174.1	132.1	25
Atrazine-desethyl	188.1	146.1	23
	188.1	110.1	31
Azinphos ethyl	346.0	132.1	-20
	346.0	160.1	-11
Azinphos-methyl	317.9	159.9	6
	317.9	260.9	6
Azoxystrobin	404.0	329.0	30
	404.0	372.0	15
Benalaxyl	326.2	148.1	-23
	326.2	208.0	-17
Benfuracarb	411.2	194.9	-25
	411.2	252.0	-15
Bensulfuron-methyl	411.1	149.0	22
	411.1	182.0	20
Bentazone	238.9	175.0	18
	238.9	196.9	19
Bifenazate	301.0	198	11
	301.0	170	22
Bifenthrin	440.2	166.3	47
	440.2	181.3	13
Boscalid	342.9	139.9	20
	342.9	307.0	20
Bromoxynil	275.8	78.7	17
	275.8	80.6	17
Bromuconazole	376.0	70.1	25

	376.0	158.9	35
Bupirimate	317.0	108.0	28
	317.0	166.0	28
Buprofezin	306.1	57.4	20
	306.1	201.0	12
Cadusafos	271.1	131.0	22
	271.1	159.0	16
Carbaryl	202.0	117.0	28
	202.0	145.0	22
Carbendazim	192.1	132.1	28
	192.1	160.1	18
Carbofuran	222.1	123.0	16
	222.1	165.1	13
Carbosulfan	381.0	118.0	15
	381.0	160.0	10
Carboxin	236.0	87.0	22
	236.0	143.0	16
Chlorantraniliprole	484.0	452.9	-17
	482.0	284.0	-14
Chlorbromuron	295.1	206.0	-20
	293.1	182.1	-17
Chlorfluazuron	539.8	158.0	20
	539.8	382.9	20
Chloridazon	222.0	77.0	30
	222.0	92.0	30
Chlorpyrifos	350.0	197.8	25
	350.0	97.1	32
Chlorpyrifos-methyl	322.1	125.1	35
	322.1	290.0	20
Chlorsulfuron	358.0	141.0	16
	358.0	167.0	18
Clethodim	360.0	164.0	18
	360.0	268.1	12
Clodinafop	350.0	91.0	32
	350.0	266.0	16
Clofentezine	303.0	102.0	35
	303.0	138.0	22
Cyantraniliprole	475.0	286.0	-17
	475.0	444.0	-16
Cyazofamid	325.2	108.1	14
	325.1	261.1	10
Cycloate	216.0	134.0	6
	216.0	154.0	4
Cyflumetofen	465.2	173.0	24
	465.2	249.0	16
Cymoxanil	199.0	111.0	18
	199.0	128.0	8
Cypermethrin	181.0	152.0	25
	181.0	127.0	35
Cyproconazole	292.2	70.2	18

	292.2	125.1	24
Cyprodinil	226.0	93.0	33
	226.0	108.0	25
Dazomet	162.9	89.8	9
	162.9	119.9	14
Deltamethrin	505.9	93.2	46
	505.9	280.9	12
Demeton-S-methyl	231.1	61.2	30
	231.1	81.9	10
Demeton-S-methyl-sulfone	263.0	121.0	17
	263.0	169.0	17
Demeton-S-methyl-sulfoxide	247.0	109.0	25
	247.0	169.0	14
Desmedipham	301.0	136.0	22
	301.0	182.0	10
Diafenthiuron	385.4	278.3	33
	385.4	329.3	19
Diazinon	305.0	97.0	35
	305.0	169.0	17
Dichlofluanid	333.0	123.0	24
	333.0	224.0	10
Dichlorvos	221.0	79.0	34
	221.0	109.0	22
Dicrotophos	238.0	112.0	10
	238.0	193.0	10
Diethofencarb	268.0	124.0	40
	268.0	226.0	10
Difenoconazole	406.0	111.1	60
	406.0	251.1	25
Diflubenzuron	309.0	155.9	11
	309.0	289.0	10
Dimethenamid	276.0	168.0	26
	276.0	244.0	14
Dimethoate	230.1	125.0	20
	230.1	199.0	10
Dimethomorph	388.1	165.0	30
	388.1	300.9	20
Dinocap	295.1	209.0	31
	295.1	193.4	28
Dinotefuran	203.1	129.1	-15
	203.1	157.2	-12
Dithianon	296.8	263.9	14
	296.8	238.0	18
Diuron	233.0	46.3	14
	233.0	72.1	18
Dodine	228.0	57.1	23
	228.0	60.1	23
Epoxiconazole	330.0	101.0	50
	330.0	121.0	22
Ethiofencarb	226.1	107.0	17

	226.1	164.0	8
Ethofumesate	287.1	121.1	15
	287.1	259.1	10
Etofenprox	394.3	106.9	43
	394.3	177.0	15
Etoxazole	360.5	141.2	28
	360.5	177.3	20
Famoxadone	392.2	238.0	20
	392.2	331.1	10
Fenamidone	312.1	92.0	25
	312.1	236.1	14
Fenarimol	331.0	81.0	34
	331.0	268.0	22
Fenazaquin	307.2	57.2	25
	307.2	161.0	19
Fenbuconazole	337.0	70.1	20
	337.0	125.0	36
Fenhexamid	302.1	55.3	38
	302.1	97.2	22
Fenoxaprop-P-ethyl	362.1	121.0	27
	362.1	287.9	18
Fenoxycarb	302.1	88.0	20
	302.1	116.1	11
Fenpropathrin	350.1	97.0	34
	350.1	125.0	14
Fenpyroximate	422.2	138.1	32
	422.2	366.1	15
Fenthion	279.1	169.1	16
	279.1	247.1	13
Flonicamid	230.0	202.9	18
	230.1	148.1	28
Fluazinam	463.0	398.0	20
	463.0	415.9	19
Flubendiamide	683.0	408	-11
	700.0	408	-21
Fludioxonil	247.0	180.0	28
	247.0	126.0	35
Flufenoxuron	489.1	141.0	46
	489.1	158.0	22
Fluopicolide	382.9	173.0	24
	382.9	144.9	55
Fluopyram	397.1	172.9	33
	397.1	207.9	23
Flupyradifurone	289.1	126.1	-23
	289.1	90.0	-23
Flutriafol	302.1	70.2	18
	302.1	123.1	29
Fluxapyroxad	382.0	362.0	10
	382.0	342.0	20
Fonofos	247.1	109.0	20

	247.1	137.0	10
Formetanate	222.0	46.0	26
	222.0	165.0	15
Fosthiazate	284.1	104	22
	284.1	227.9	12
Furathiocarb	383.2	194.9	18
	383.2	252.0	12
Haloxyfop	434.0	91.0	34
	434.0	316.0	20
Haloxyfop-P-methyl	376.0	316.1	32
	376.0	288.0	25
Heptenophos	251.0	125.0	14
	251.0	127.0	22
Hexaconazole	314.0	70.1	28
	314.0	159.0	28
Hexaflumuron	459.0	276.0	19
	459.0	439.0	12
Hexythiazox	353.0	168.1	26
	353.0	228.1	14
Imazalil	297.0	69.0	22
	297.0	159.0	22
Imidacloprid	256.1	175.1	20
	256.1	209.1	15
Indoxacarb	528.1	203.0	36
	528.1	150.0	16
Isoprocarb	210.8	95.0	-22
	210.8	137.0	-16
Isopyrazam	360.2	244.1	-27
	360.2	340.3	-14
Ioxynil	369.8	127.1	31
	369.8	215.1	30
Iprodione	330.0	245.0	10
	330.0	288.0	5
Iprovalicarb	320.8	119.1	-23
	320.8	203.1	-11
Kresoxim-methyl	314.0	116.0	25
	314.0	206.0	40
Lambda-cyhalothrin	467.0	141.0	45
	467.0	225.0	10
Lenacil	235.2	136.1	32
	235.2	153.1	16
Linuron	249.1	160.1	18
	249.1	181.1	16
Lufenuron	509.2	175.0	40
	509.2	326.1	22
Malathion	331.0	99.0	24
	331.0	127.0	12
Malaoxon	315.0	98.9	24
	315.0	127.0	12
Mandipropamid	412.0	328.0	-17

	412.0	356.1	-12
Metaflumizone	505.0	302.0	10
	505.0	117.0	48
Metalaxyl	280.3	192.4	19
	280.3	220.4	14
Metamitron	203.1	104.0	22
	203.1	175.1	16
Methacrifos	241.1	125.0	20
	241.1	209.1	8
Methamidophos	142.0	93.9	13
	142.0	124.9	13
Methiocarb	226.0	169.0	22
	226.0	164.0	10
Methoxyfenozide	369.1	149.0	-21
	369.1	133.1	-33
Metrafenone	409.1	209.0	-16
	409.1	226.9	-17
Metribuzin	215.0	89.0	20
	215.0	131.0	18
Mevinphos	225.1	127.1	15
	225.1	193.1	8
Monocrotophos	224.1	98.1	12
	224.1	127.1	16
Monolinuron	215.0	99.0	34
	215.0	126.0	22
Myclobutanil	289.1	70.2	18
	289.1	125.1	32
Nicosulfuron	411.0	106.0	32
	411.0	182.0	22
Nuarimol	315.0	81.1	28
	315.0	252.0	22
Omethoate	214.1	125.1	22
	214.1	183.1	11
Oxadixyl	279.0	132.0	34
	279.0	219.0	10
Oxamyl	237.0	72.0	10
	237.0	90.0	10
Oxyfluorfen	362.0	237.0	20
	362.0	316.0	5
Paraoxon-ethyl	276.4	174.3	25
	276.4	220.3	14
Penconazole	284.0	70.1	16
	284.0	159.0	34
Pendimethalin	282.2	194.1	17
	282.2	212.2	10
Phenmedipham	301.0	136.0	22
	301.0	168.0	10
Phenthoate	321.0	135.0	20
	321.0	163.0	12
Phorate	261.0	74.9	8

	261.0	170.9	12
Phosalone	367.9	110.9	42
	367.9	181.9	14
Phosphamidon	300.1	127.1	25
	300.1	174.1	14
Phosmet	318.0	77.0	46
	318.0	160.0	22
Pirimicarb	239.1	72.0	18
	239.1	182.1	15
Pirimiphos-ethyl	334.2	198.1	19
	334.2	182.1	19
Pirimiphos-methyl	306.0	108.0	31
	306.0	164.0	19
Prochloraz	376.2	70.1	34
	376.2	308.1	11
Profenofos	373.0	97.0	35
	373.0	303.0	15
Profoxydim	466.1	180.1	26
	466.1	280.1	24
Promecarb	208.1	109.0	15
	208.1	151.0	9
Prometryn	242.0	158.0	25
	242.0	200.1	17
Propamocarb	189.1	102.0	17
	189.1	144.0	22
Propaquizafop	444.2	100.0	20
	444.2	163.1	60
Propargite	368.4	57.1	20
	368.4	231.3	8
Propazine	230.2	146.1	24
	230.2	188.1	18
Propiconazole	342.0	69.0	22
	342.0	159.0	34
Propoxur	210.0	111.0	16
	210.0	168.0	10
Propyzamide	256.1	173.0	23
	256.1	190.0	16
Prosulfuron	418.1	139.1	22
	418.1	252.1	14
Prothiofos	345.1	133.0	56
	345.1	241.0	18
Pymetrozine	218.0	79.0	30
	218.0	105.0	20
Pyraclostrobin	388.0	194.0	-14
	388.0	162.9	-28
Pyrazophos	374.0	194.0	33
	374.0	222.0	17
Pyridaben	365.1	147.1	24
	365.1	309.1	12
Pyridaphenthion	341.0	92.0	34

	341.0	189.0	22
Pyridate	379.0	207.0	18
	379.0	351.1	10
Pyrimethanil	199.8	107.1	28
	199.8	82.2	30
Pyriproxyfen	322.1	96.0	14
	322.1	227.1	14
Rimsulfuron	431.9	182.1	22
	431.9	325.1	14
Sethoxydim	328.0	178.0	22
	328.0	282.0	10
Simazine	202.0	96.0	22
	202.0	124.0	16
Spinosyn A	732.6	98.1	59
	732.6	142.0	31
Spinosyn D	746.5	98.1	53
	746.5	142.0	31
Spinetoram	748.4	142.1	-37
	748.4	98.2	-55
Spirodiclofen	411.2	71.2	22
	413.1	315.1	14
Spiromesifen	371.1	273.2	12
	371.1	255.2	26
Spirotetramat	374.1	302.1	-18
	374.1	216	-37
Spirotetramat-enol	302.1	216	-30
	302.1	116.9	-42
Spiroxamine	298.0	100.0	32
	298.0	144.0	20
Sulfoxaflor	278.0	174.0	-10
	278.0	105.1	-13
Tau-fluvalinate	503.0	181.0	30
	503.0	208.0	5
Tebuconazole	308.0	70.1	22
	308.0	125.0	40
Tebufenozide	353.1	133.0	20
	353.1	297.1	8
Tebufenpyrad	334.0	117.0	34
	334.0	145.0	28
Teflubenzuron	379.0	339	11
	379.0	358.9	6
Tepraloxydim	342.1	166.1	20
	342.1	250.1	12
Terbufos	289.0	57.2	22
	289.0	103.0	8
Terbutryn	242.0	68.0	45
	242.0	186.0	13
Tetraconazole	372.0	70.1	20
	372.0	159.0	30
Thiabendazole	202.0	131.0	30

	202.0	175.0	25
Thiacloprid	253.0	90.1	40
	253.0	126.0	20
Thiamethoxam	292.0	132.0	22
	292.0	211.2	12
Thifensulfuron-methyl	388.0	56.0	40
	388.0	167.0	15
Thiobencarb	258.2	89.1	48
	258.2	125.1	18
Thiodicarb	355.0	87.9	16
	355.0	107.9	16
Thiophanate-methyl	343.0	93.0	46
	343.0	151.0	22
Tolyfluanid	364.0	137.0	25
	364.0	238.0	1
Tralkoxydim	330.2	138.1	19
	330.2	284.3	13
Triadimefon	294.1	69.3	20
	294.1	197.2	15
Triadimenol	296.1	70.2	10
	296.1	99.1	15
Triallate	304.0	86.0	18
	304.0	142.9	28
Triasulfuron	402.0	141.0	20
	402.0	167.1	17
Trichlorfon	257.0	79.0	30
	257.0	109.0	18
Trifloxystrobin	409.0	145.0	40
	409.0	186.0	16
Triflumizole	346.0	73.1	10
	346.0	277.9	10
Triflumuron	356.9	154.0	13
	356.8	84.7	45
Triticonazole	318.1	70.1	16
	318.1	124.9	35
Valifenalate	399.2	154.9	40
	401.2	116.0	29
Vamidothion	288.0	118.1	28
	288.0	146.0	10
Zoxamide	336.1	186.9	-23
	336.1	158.9	-46

Table S2. Definitions and scoring criteria for the indices used in pesticide residue risk ranking.

Categories	Definition	Score	Definition	Score	Definition	Score	Definition	Score
A: Toxicity (LD_{50})	Low toxicity	2	Moderate toxicity	3	Highly toxicity	4	Rank poison	5
B: Toxic effect (ADI)	＞0.01	0	0.0001–0.01	1	0.000001–0.0001	2	＜0.000001	3
C: Proportion of diet (%)	＜2.5	0	2.5–20	1	20–50	2	50–100	3
D: Frequency of pesticide (%)	＜2.5	0	2.5–20	1	20–50	2	50–100	3
E: High-exposure crowd	nothing	0	few	1	mainly	2	indeterminacy	3
F: Residual level	ND	1	＜1MRL	2	1–10MRL	3	≥10MRL	4

Table S3. Scores assigned to indices A–F for the pesticide residue risk-ranking system.

Pesticide	Nature of the hazard, A	Toxicological potency (ADI), B	Dietary contribution, C				Pesticide application frequency, D				High-exposure groups, E	Residue occurrence relative to MRLs, F			
			**Pepper**	**Cucumber**	**Tomato**	**Courgette**	**Pepper**	**Cucumber**	**Tomato**	**Courgette**		**Pepper**	**Cucumber**	**Tomato**	**Courgette**
Acetamiprid	3	1	1	1	1	–	1	1	0	–	3	1.32	1.79	1.16	–
Ametoctradin	2	0	–	1	1	–	–	1	1	–	3		1.03	1.03	–
Azoxystrobin	2	0	1	1	1	–	0	1	1	–	3	1.07	1.01	1.08	–
Boscalid	2	0	1	–	1	0	1	–	1	1	3	1.27	–	1.11	1.04
Chlorantraniliprole	2	0	1	–	1	–	1	–	1	–	3	1.05	–	1.11	–
Clofentezine*	2	0	1	–	–	–	0	–	–	–	3	1.01	–	–	–
Cyantraniliprole	2	0	–	–	1	–	–	–	1	–	3	–	–	1.01	–
Difenoconazole	3	1	1	–	–	–	1	–	–	–	3	1.01	–	–	–
Dimethomorph*	2	0	–	1	–	–	–	1	–	–	3	–	1.01	–	–
Famoxadone*	2	1	–	1	–	–	–	1	–	–	3	–	1.03	–	–
Flonicamid	3	0	1	1	1	–	0	1	0	–	3	1.19	1.01	1.01	–
Fluopicolide	2	0	–	1	–	–	–	1	–	–	3	–	1.01	–	–
Flupyradifurone	3	0	1		–	–	0		–	–	3	1.03	–	–	–
Metaflumizone*	2	1	–	–	1	–	–	–	1	–	3	–	–	1.03	–
Metalaxyl	3	0	–	1	–	–	–	1	–	–	3	–	1.17	–	–
Methoxyfenozide*	2	0	–	–	1	–	–	–	1	–	3	–	–	1	–
Metrafenone	2	0	–	–	–	0	–	–	–	1	3	–	–	–	1.01
Penconazole	2	0	–	–	–	0	–	–	–	1	3	–	–	–	1.01
Propamocarb	2	0	–	1	–	0	–	1	–	1	3	–	1.16	–	1.03
Pyraclostrobin	-	0	–	1	–	–	–	1	–	–	3	–	1.01	–	–
Pyridaben	3	0	1		1	–	1		1	–	3	1.01	–	1.03	–
Spirotetramat*	2	0	1		1	–	1		1	–	3	1.07	–	1.03	–
Tebuconazole	3	0	1	1	1	–	1	1	1	–	3	1.01	1.03	1.03	–

Table S4. Occurrence and concentration of pesticide residues detected in pepper, cucumber, tomato, and courgette samples.

Pesticide	Pepper			Cucumber			Tomato			Courgette		
	Positive samples, *n* (%)	Concentration (mg kg^{-1})		Positive samples, *n* (%)	Concentration (mg kg^{-1})		Positive samples, *n* (%)	Concentration (mg kg^{-1})		Positive samples, *n* (%)	Concentration (mg kg^{-1})	
		**Range**	**Mean**		**Range**	**Mean**		**Range**	**Mean**		**Range**	**Mean**
Acetamiprid	15 (20)	0.019–0.121	0.066	19 (25.3)	0.033–0.741	0.144	3 (4)	0.121–0.185	0.164	–	–	–
Ametoctradin	–	–	–	2 (2.7)	0.022–0.258	0.140	2 (2. 7)	0.02–0.046	0.033	–	–	–
Azoxystrobin	5 (6.7)	0.053–0.287	0.150	1 (1.3)	0.019	0.019	6 (8)	0.011–0.054	0.034	–	–	–
Boscalid	20 (26.7)	0.016–0.128	0.060	–	–	–	8 (10. 7)	0.036–0.246	0.077	3 (4)	0.016–0.056	0.040
Chlorantraniliprole	4 (5.3)	0.014–0.123	0.065	–	–	–	8 (10.7)	0.014–0.134	0.058	–	–	–
Clofentezine*	1 (1.3)	0.145	0.145	–	–	–	–	–	–	–	–	–
Cyantraniliprole	–	–	–	–	–	–	1 (1.3)	0.014	0.014	–	–	–
Difenoconazole	1 (1.3)	0.124	0.124	–	–	–	–	–	–	–	–	–
Dimethomorph*	–	–	–	1 (1.3)	0.029	0.029	–	–	–	–	–	–
Famoxadone*	–	–	–	2 (2.7)	0.052–0.088	0.070	–	–	–	–	–	–
Flonicamid	14 (18.7)	0.013–0.077	0.044	1 (1.3)	0.031	0.031	1 (1.3)	0.102	0.102	–	–	–
Fluopicolide	–	–	–	1 (1.3)	0.029	0.029	–	–	–	–	–	–
Flupyradifurone	2 (2.7)	0.014–0.023	0.019				–	–	–	–	–	–
Metaflumizone*	–	–	–	–	–	–	4 (5.3)	0.021–0.055	0.036	–	–	–
Metalaxyl	–	–	–	10 (13.3)	0.012–0.784	0.212	–	–	–	–	–	–
Methoxyfenozide*	–	–	–	–	–	–	2 (2. 7)	0.107–0.167	0.137	–	–	–
Metrafenone	–	–	–	–	–	–	–	–	–	1 (1.3)	0.052	0.052
Penconazole	–	–	–	–	–	–	–	–	–	1 (1.3)	0.043	0.043
Propamocarb	–	–	–	12 (16)	0.032–0.13	0.069	–	–	–	2 (2.7)	0.111–0.131	0.121
Pyraclostrobin	–	–	–	1 (1.3)	0.087	0.087	–	–	–	–	–	–
Pyridaben	1 (1.3)	0.041	0.041				1 (1.3)	0.041	0.041	–	–	–
Spirotetramat*	5 (6.7)	0.069–0.385	0.167				6 (8)	0.016–0.639	0.139	–	–	–
Tebuconazole	1 (1.3)	0.049	0.049	2 (2.7)	0.033–0.038	0.036	2 (2.7)	0.049–0.057	0.053	–	–	–

*Not approved in the EU.

Table S5. Chronic and acute hazard quotients (*HQc* and *HQa*) for pesticide residues detected in pepper, cucumber, tomato, and courgette samples for adults and children.

Pesticide	Pepper				Cucumber				Tomato				Courgette			
	Adults		Children		Adults		Children		Adults		Children		Adults		Children	
	***HQc* (UB)**	***HQa***	***HQc* (UB)**	***HQa***	***HQc* (UB)**	***HQa***	***HQc* (UB)**	***HQa***	***HQc* (UB)**	***HQa***	***HQc* (UB)**	***HQa***	***HQc* (UB)**	***HQa***	***HQc* (UB)**	***HQa***
Acetamiprid	0.0053	0.2822	0.0065	0.7642	0.0053	3.5475	0.0065	5.8447	0.0157	0.5048	0.0192	1.4073	–	–	–	–
Ametoctradin	–	–	–	–	8.1×10^{-7}	0.0006	9.9×10^{-7}	0.0010	5.2×10^{-6}	0.0001	6.3×10^{-6}	0.0002	–	–	–	–
Azoxystrobin	0.0001	0.0167	0.0001	0.0453	3.0×10^{-5}	0.0023	3.7×10^{-5}	0.0037	0.0003	0.0037	0.0004	0.0103	–	–	–	–
Boscalid	0.0007	0.0373	0.0009	0.1010	–	–	–	–	0.0021	0.0839	0.0025	0.2339	0.0001	0.0044	0.0001	0.0048
Chlorantraniliprole	1.0×10^{-5}	0.0009	1.3×10^{-5}	0.0025	–	–	–	–	4.7×10^{-5}	0.0012	0.0001	0.0033	–	–	–	–
Clofentezine	0.0009	0.0995	0.0011	0.2693	–	–	–	–	–				–	–	–	–
Cyantraniliprole	–				–	–	–	–	0.0049	0.0191	0.0060	0.0532	–	–	–	–
Difenoconazole	0.0015	0.0090	0.0018	0.0245	–	–	–	–	–	–	–	–	–	–	–	–
Dimethomorph	–	–	–	–	0.0001	0.0012	0.0002	0.0019	–	–	–	–	–	–	–	–
Famoxadone	–	–	–	–	0.0012	0.0211	0.0014	0.0347	–	–	–	–	–	–	–	–
Flonicamid	0.0008	0.0359	0.0010	0.0973	0.0002	0.0297	0.0003	0.0489	0.0022	0.0557	0.0027	0.1552	–	–	–	–
Fluopicolide	–				0.0001	0.0039	0.0001	0.0064	–	–	–	–	–	–	–	–
Flupyradifurone	0.0002	0.0018	0.0002	0.0048					–	–	–	–	–	–	–	–
Metaflumizone	–	–	–	–	–				0.0006	0.0075	0.0007	0.0209	–	–	–	–
Metalaxyl	–	–	–	–	0.0003	0.0375	0.0003	0.0618	–	–	–	–	–	–	–	–
Methoxyfenozide	–	–	–	–	–				0.0065	0.0175	0.0079	0.0489	–	–	–	–
Metrafenone	–	–	–	–	–				–	–	–	–	8.4×10^{-6}	0.0006	1.0×10^{-5}	0.0007
Penconazole	–	–	–	–	–				–	–	–	–	0.0001	0.0003	0.0001	0.0003
Propamocarb	–	–	–	–	4.0×10^{-5}	0.0031	4.9×10^{-5}	0.0051	–	–	–	–	8.9×10^{-6}	0.0004	1.1×10^{-5}	0.0004
Pyraclostrobin	–	–	–	–	0.0002	0.0694	0.0003	0.1144	–	–	–	–	–	–	–	–
Pyridaben	0.0013	0.0096	0.0016	0.0259	–	–	–	–	0.0051	0.0112	0.0062	0.0312	–	–	–	–
Spirotetramat	0.0005	0.0045	0.0006	0.0122	–	–	–	–	0.0020	0.0087	0.0024	0.0243	–	–	–	–
Tebuconazole	0.0004	0.0190	0.0005	0.0516	0.0002	0.0303	0.0003	0.0500	0.0018	0.0259	0.0022	0.0723	–	–	–	–

[a]UB: Upper bound (results below the LOQ were replaced with the value of LOQ).
